# Supplementary material for: Synthesis and Chemopreventive Potential of 5-FU/Genistein Hybrids on Colorectal Cancer Cells
Source: Pharmaceuticals (Basel). 2022 Oct 21;15(10):1299. doi: 10.3390/ph15101299 (PMC9606943; doi:10.3390/ph15101299)
Supplement: Supplementary file 1 [file pharmaceuticals-15-01299-s001.zip › pharmaceuticals-1868305-Supplementary .pdf]

Supplementary Data
Synthesis and Chemopreventive Potential of 5-FU/Genistein Hybrids on Colorectal Cancer Cells

Gustavo Moreno-Q [1,†,] Wilson Castrillón-L [1,†], Angie Herrera-R [1,2,*], Andrés F. Yepes [1], Jorge Quintero-Saumeth[1], Wilson Cardona-G [1*]

[1] Chemistry of Colombian Plants group, Institute of Chemistry, Faculty of Exact and Natural Sciences, University of Antioquia, Calle 70 No. 52–21, A.A 1226, Medellín, Colombia;

[2] Medical and Experimental Mycology Group, Corporación para Investigaciones Biológicas, 050034, Medellín, Colombia;

***** Correspondence: angie.herrerar@udea.edu.co (A.H.-R.); wilson.cardona1@udea.edu.co (W.C.-G.

^{1}H, ^{13}C NMR and MS spectra	**Figure S1**
HPLC analysis	**Figure S2**

Figure S1. ^{1}H, ^{13}C NMR and MS spectra

5-fluoro-1-((1-(3-((5-hydroxy-3-(4-hydroxyphenyl)-4-oxo-4H-chromen-7-yl)oxy)ethyl)-1H-1,2,3-triazol-4-yl)methyl)pyrimidine-2,4(1H,3H)-dione (4a)

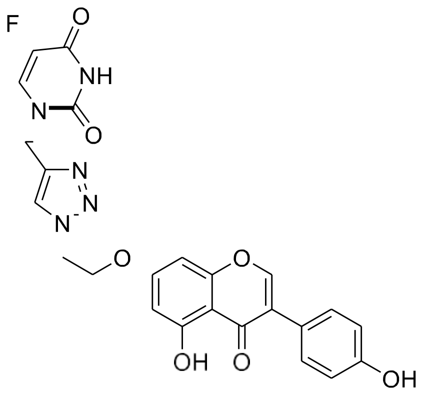
F
O
NH
N
O
N
N
N
O
O
OH
O
OH

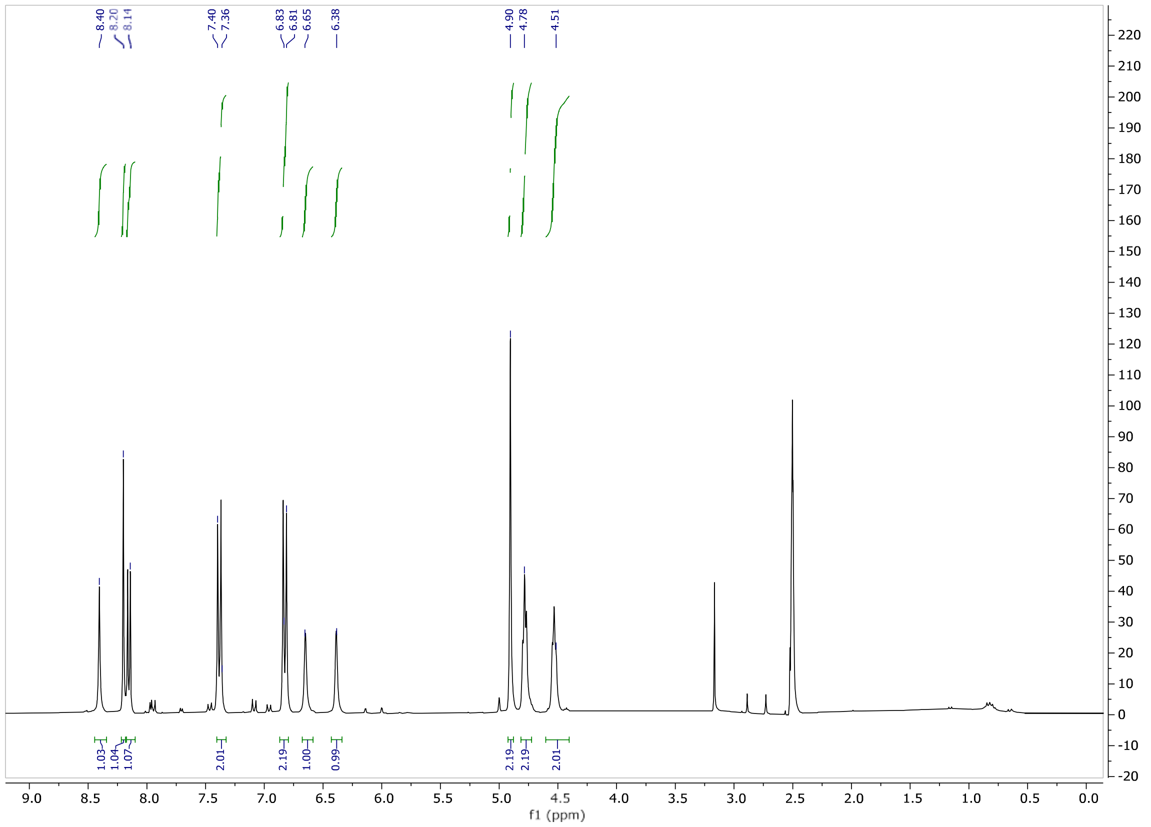
8.40
8.20
8.14
7.40
7.36
6.83
6.81
6.65
6.38
4.90
4.78
4.51
1.03
1.04
1.07
2.01
2.19
1.00
0.99
2.19
2.19
2.01
f1 (ppm)

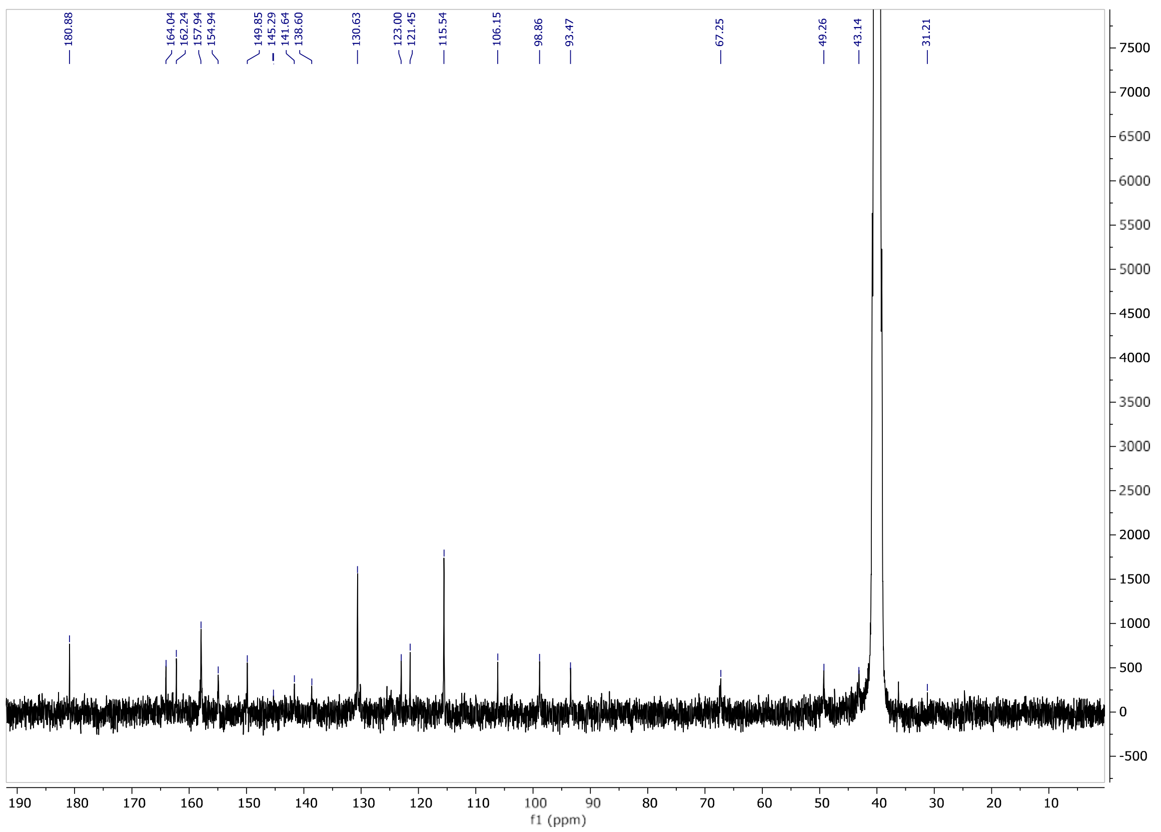

180.88
164.04
162.24
157.94
154.94
149.85
145.29
141.64
138.60
130.63
123.00
121.45
115.54
106.15
98.86
93.47
67.25
49.26
43.14
31.21
f1 (ppm)

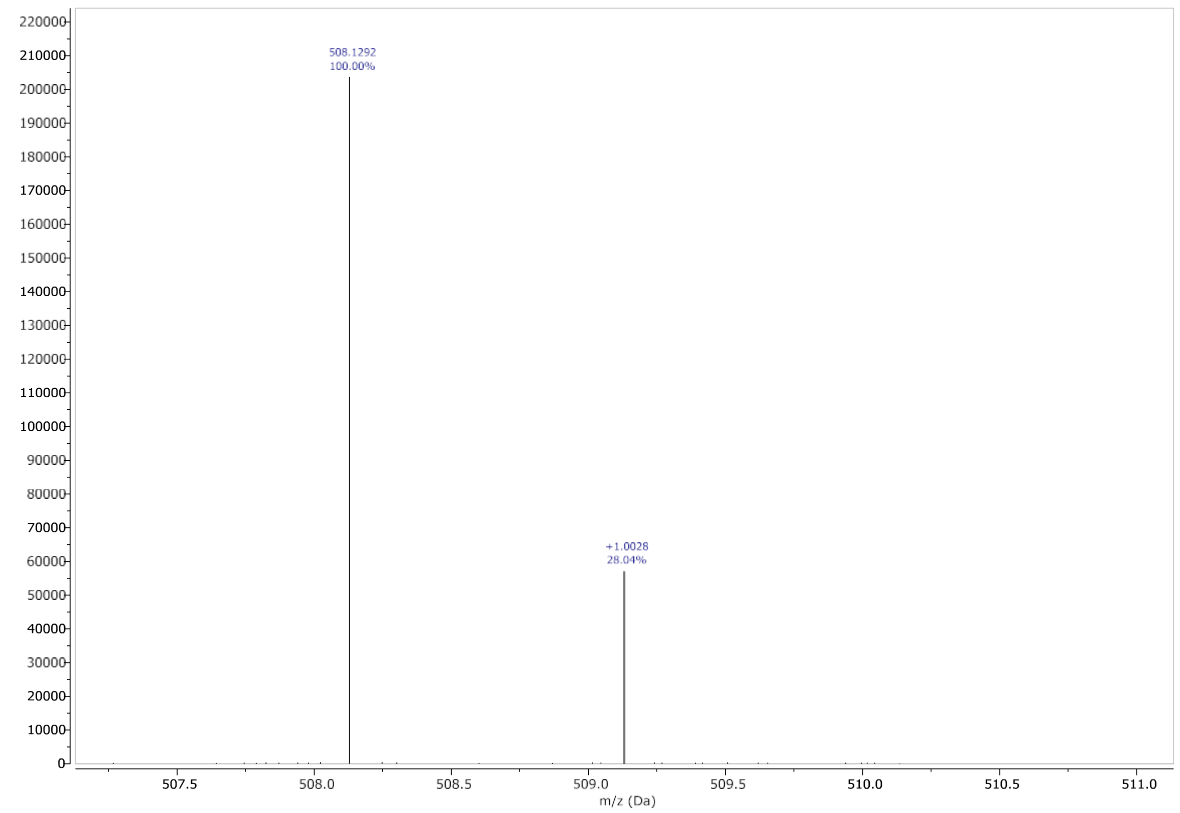

508.1292
100.00%
+1.0028
28.04%
m/z (Da)

5-fluoro-1-((1-(3-((5-hydroxy-3-(4-hydroxyphenyl)-4-oxo-4H-chromen-7-yl)oxy)propyl)-1H-1,2,3-triazol-4-yl)methyl)pyrimidine-2,4(1H,3H)-dione (4b)

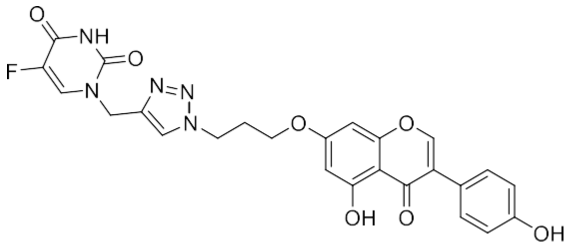

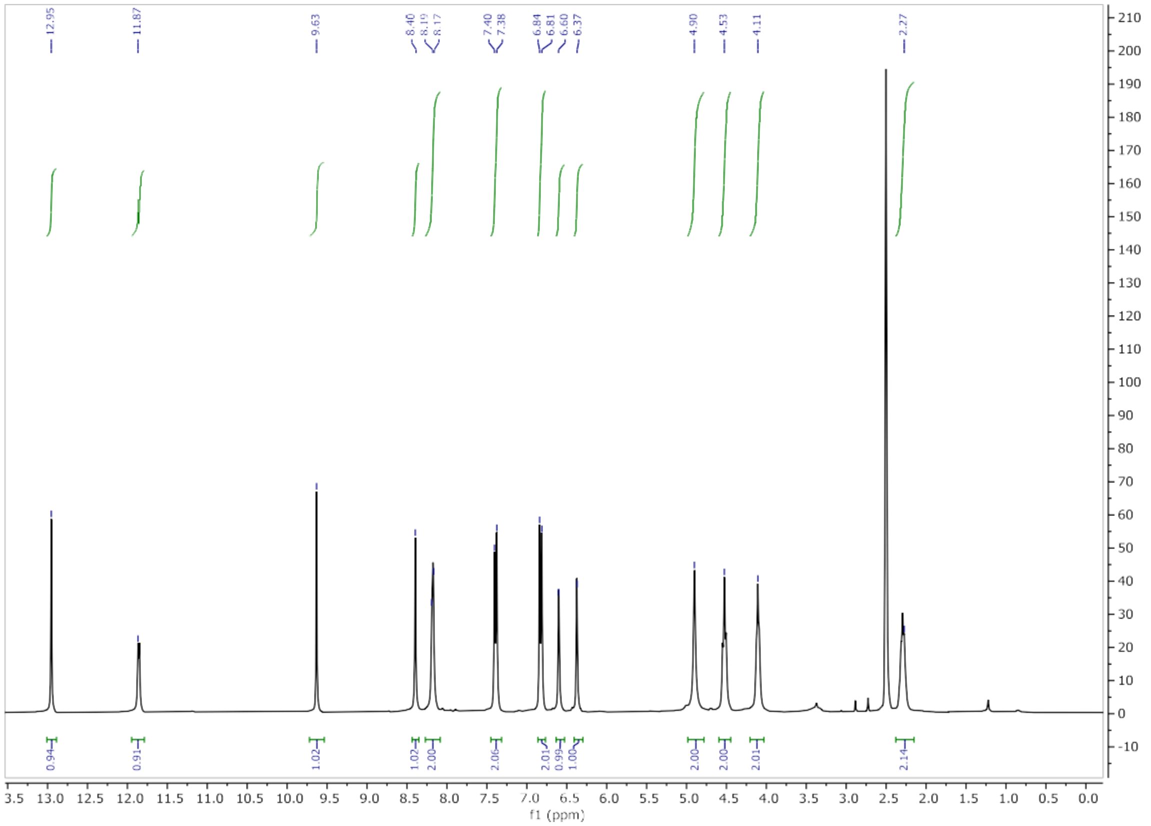

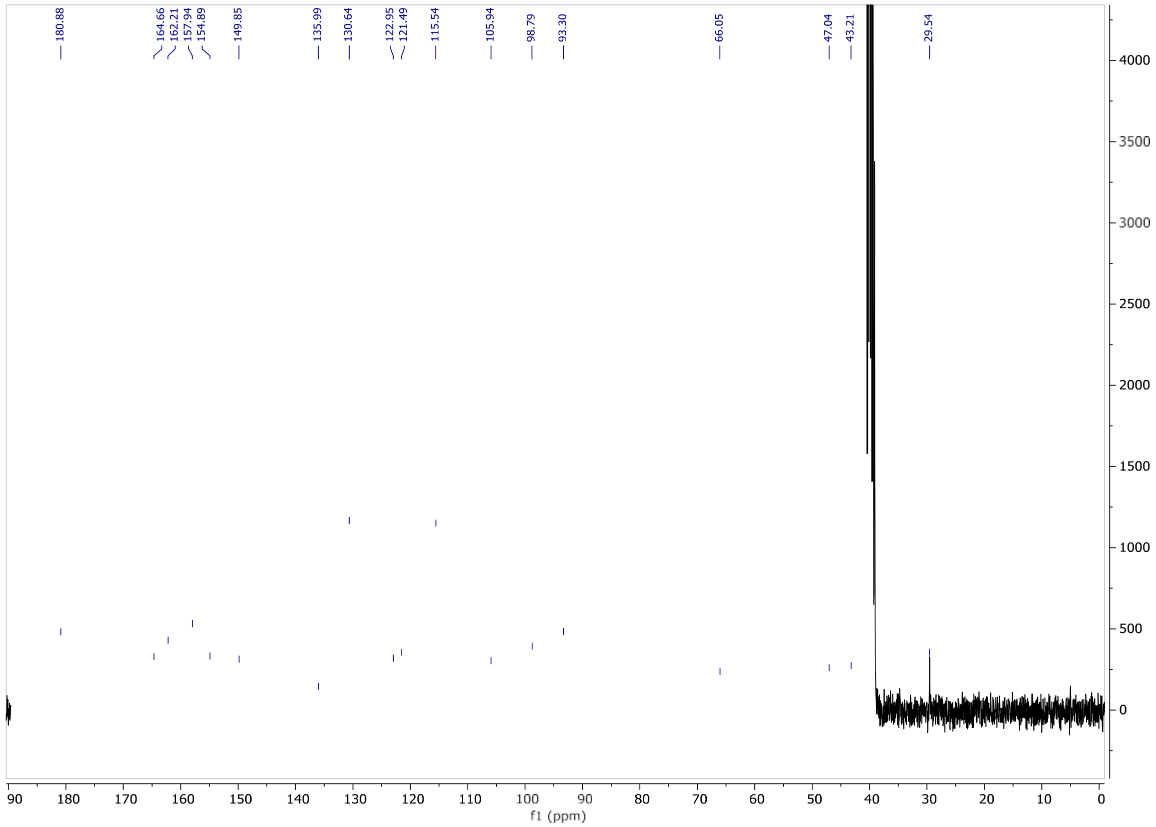

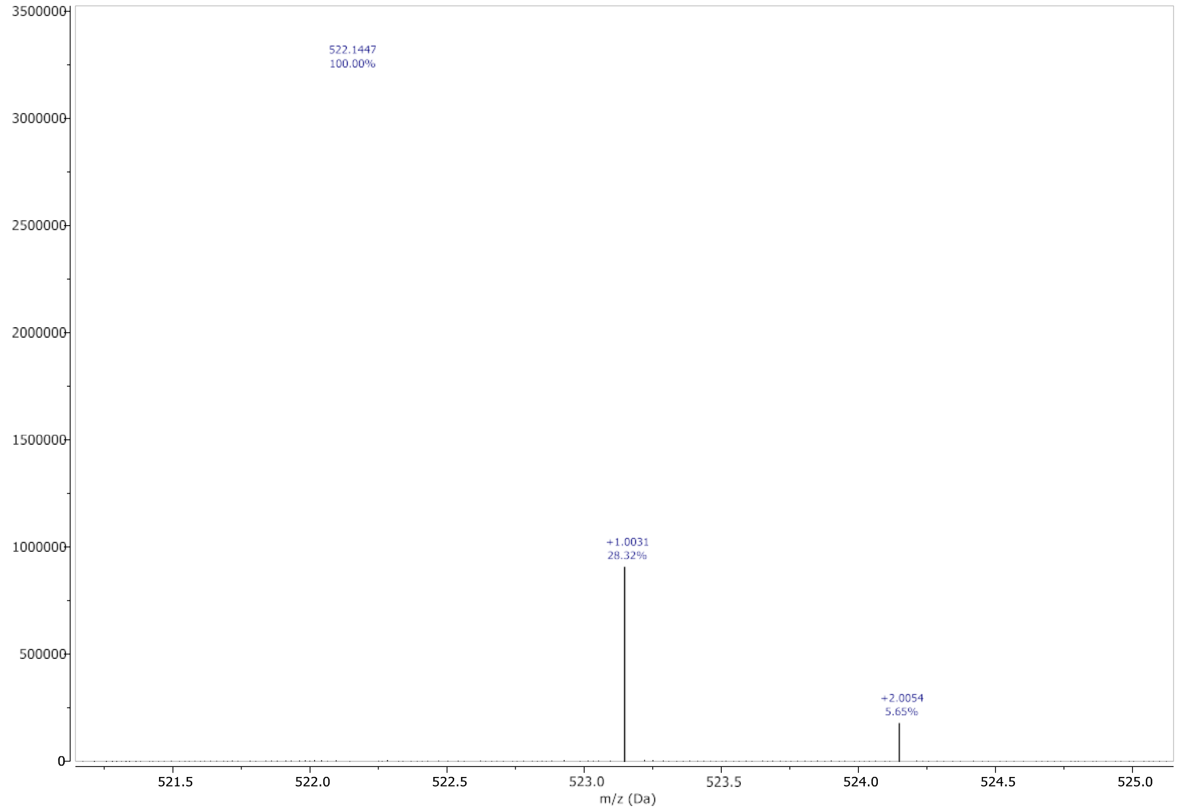

5-fluoro-1-((1-(3-((5-hydroxy-3-(4-hydroxyphenyl)-4-oxo-4H-chromen-7-yl)oxy)butyl)-1H-1,2,3-triazol-4-yl)methyl)pyrimidine-2,4(1H,3H)-dione (4c)

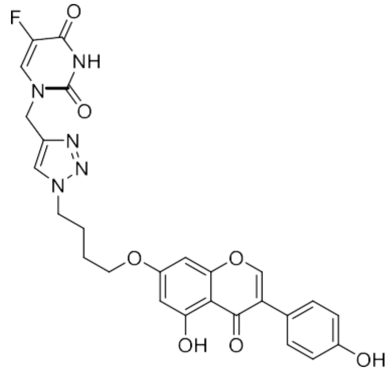
F
O
NH
N
O
N
N
N
O
O
OH
O
OH

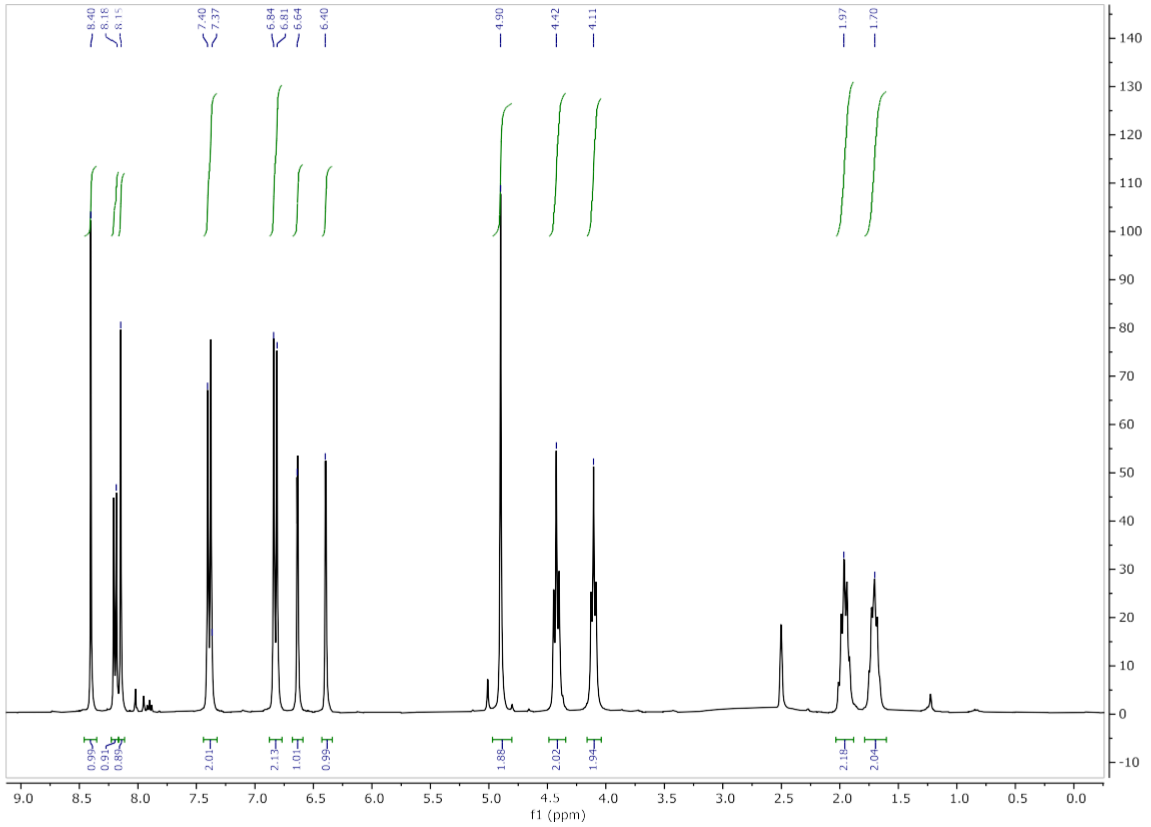
8.40
8.18
8.15
7.40
7.37
6.84
6.81
6.64
6.40
4.90
4.42
4.11
1.97
1.70
0.99
0.91
0.89
2.01
2.13
1.01
0.99
1.88
2.02
1.94
2.18
2.04
f1 (ppm)

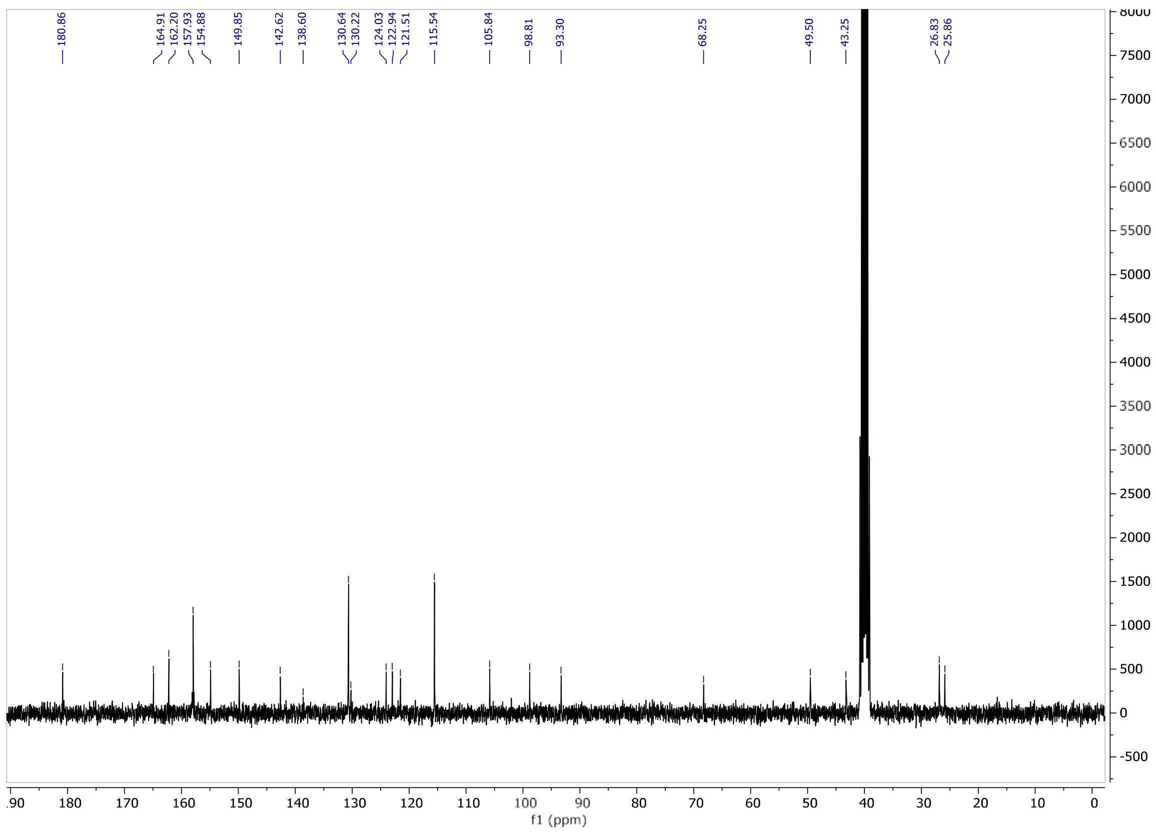

180.86
164.91
162.20
157.93
154.88
149.85
142.62
138.60
130.64
130.22
124.03
122.94
121.51
115.54
105.84
98.81
93.30
68.25
49.50
43.25
26.83
25.86
f1 (ppm)

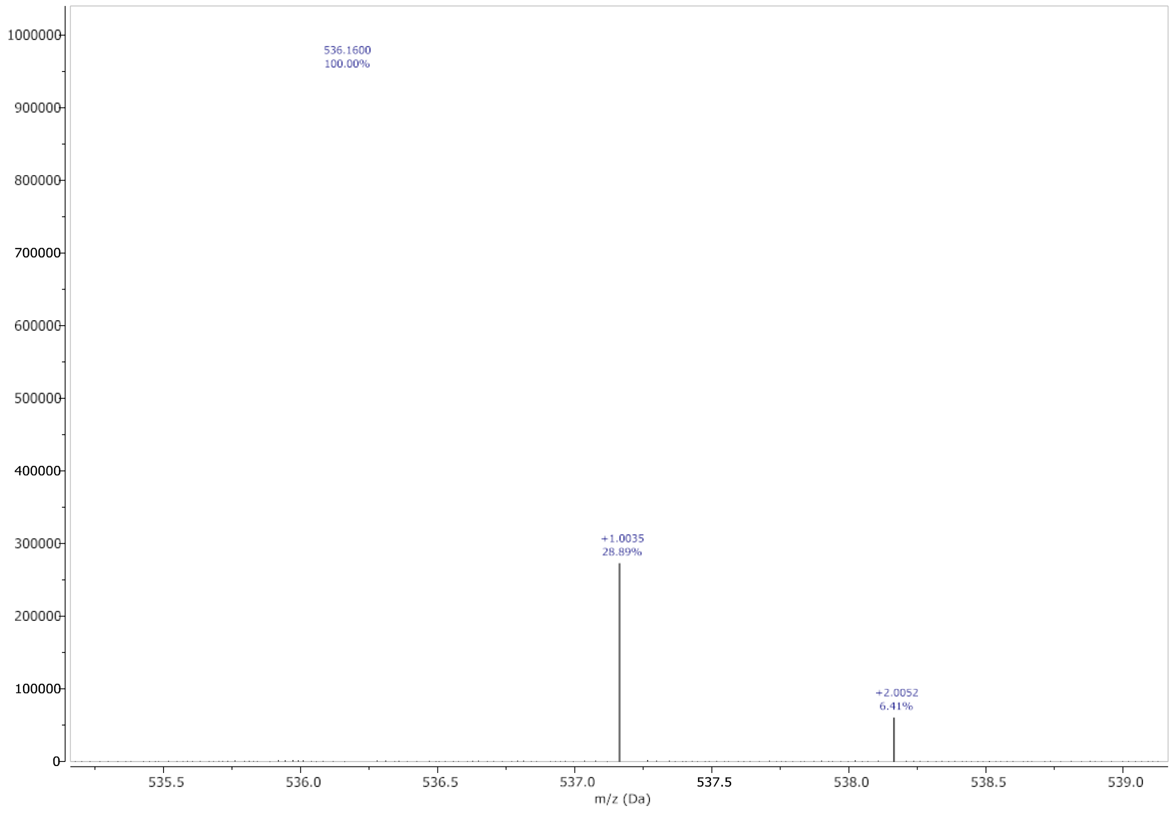

536.1600
100.00%
+1.0035
28.89%
+2.0052
6.41%
m/z (Da)

5-fluoro-1-((1-(3-((5-hydroxy-3-(4-hydroxyphenyl)-4-oxo-4H-chromen-7-yl)oxy)pentyl)-1H-1,2,3-triazol-4-yl)methyl)pyrimidine-2,4(1H,3H)-dione (4d)

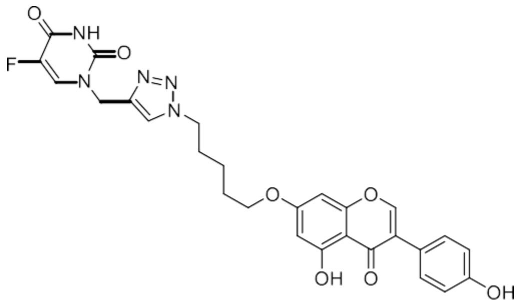

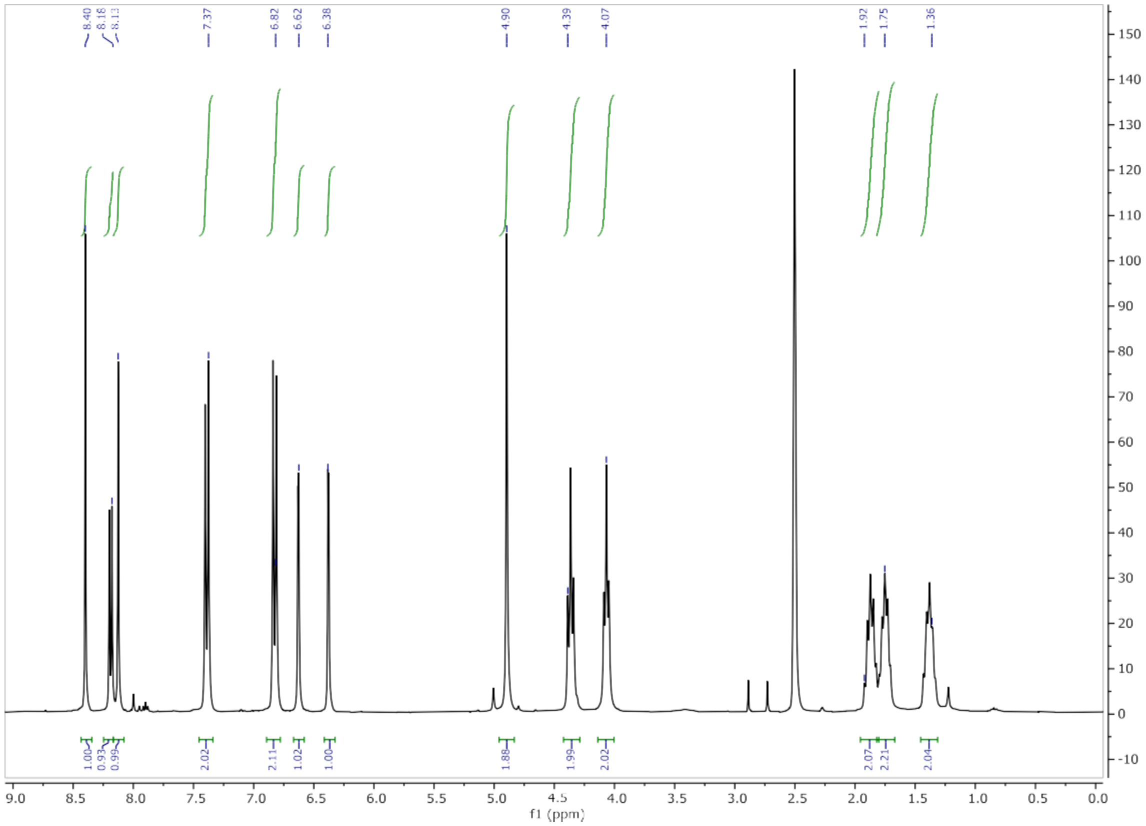

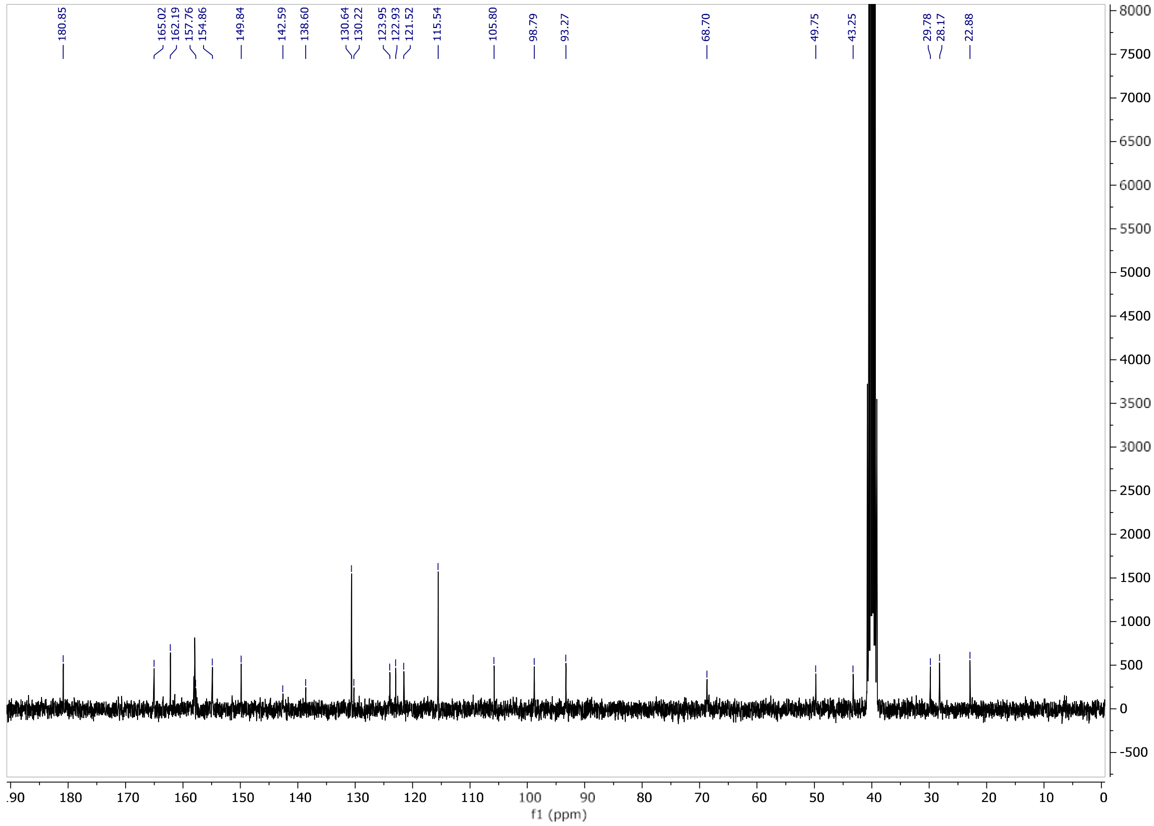

180.85
165.02
162.19
157.76
154.86
149.84
142.59
138.60
130.64
130.22
123.95
122.93
121.52
115.54
105.80
98.79
93.27
68.70
49.75
43.25
29.78
28.17
22.88
f1 (ppm)

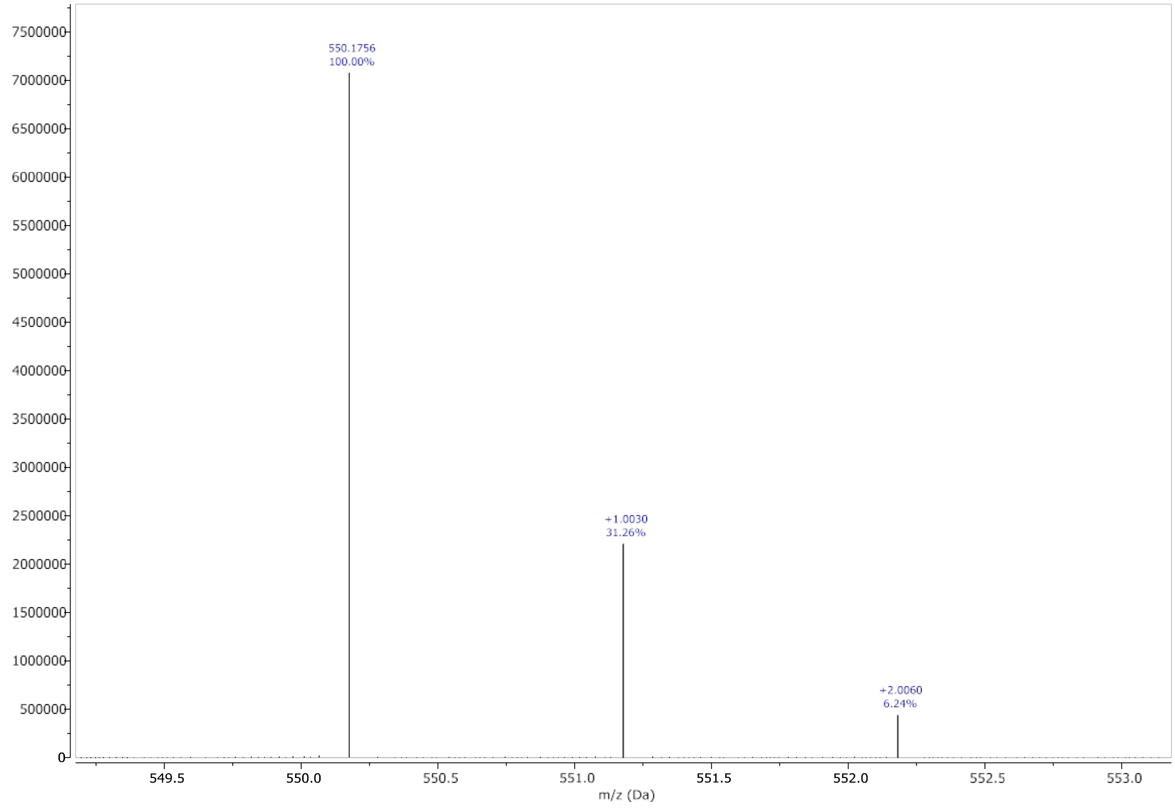

550.1756
100.00%
+1.0030
31.26%
+2.0060
6.24%
m/z (Da)

5-fluoro-1-((1-(3-((5-hydroxy-3-(4-hydroxyphenyl)-4-oxo-4H-chromen-7-yl)oxy)hexyl)-1H-1,2,3-triazol-4-yl)methyl)pyrimidine-2,4(1H,3H)-dione (4e)

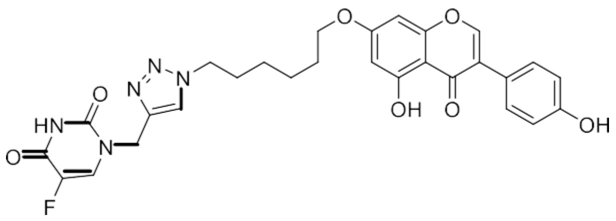

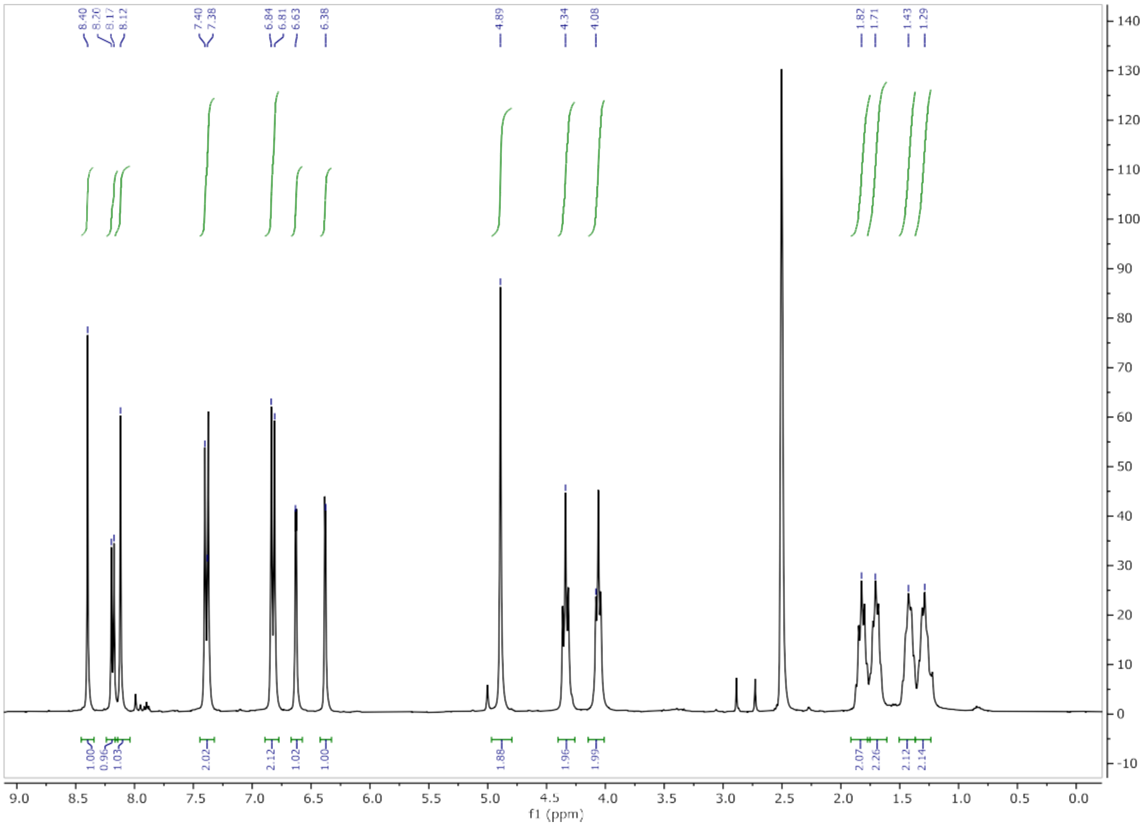

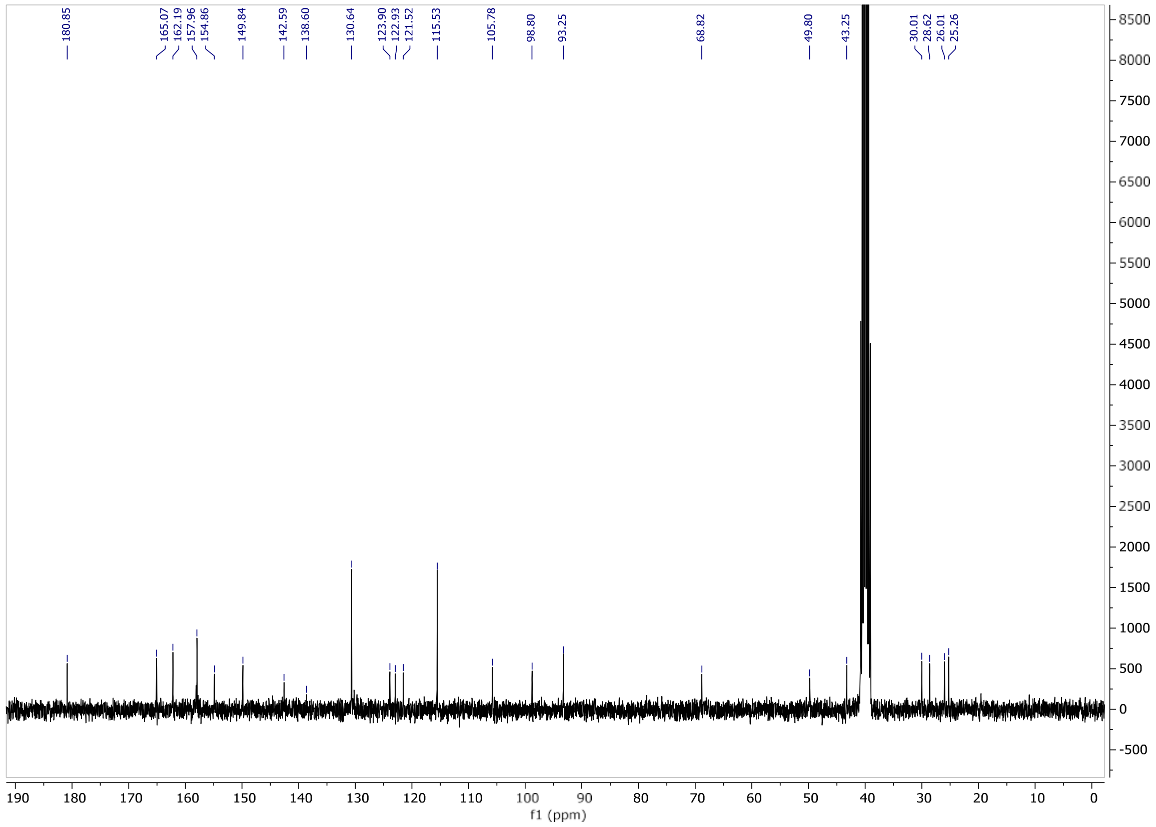

180.85
165.07
162.19
157.96
154.86
149.84
142.59
138.60
130.64
123.90
122.93
121.52
115.53
105.78
98.80
93.25
68.82
49.80
43.25
30.01
28.62
26.01
25.26
f1 (ppm)

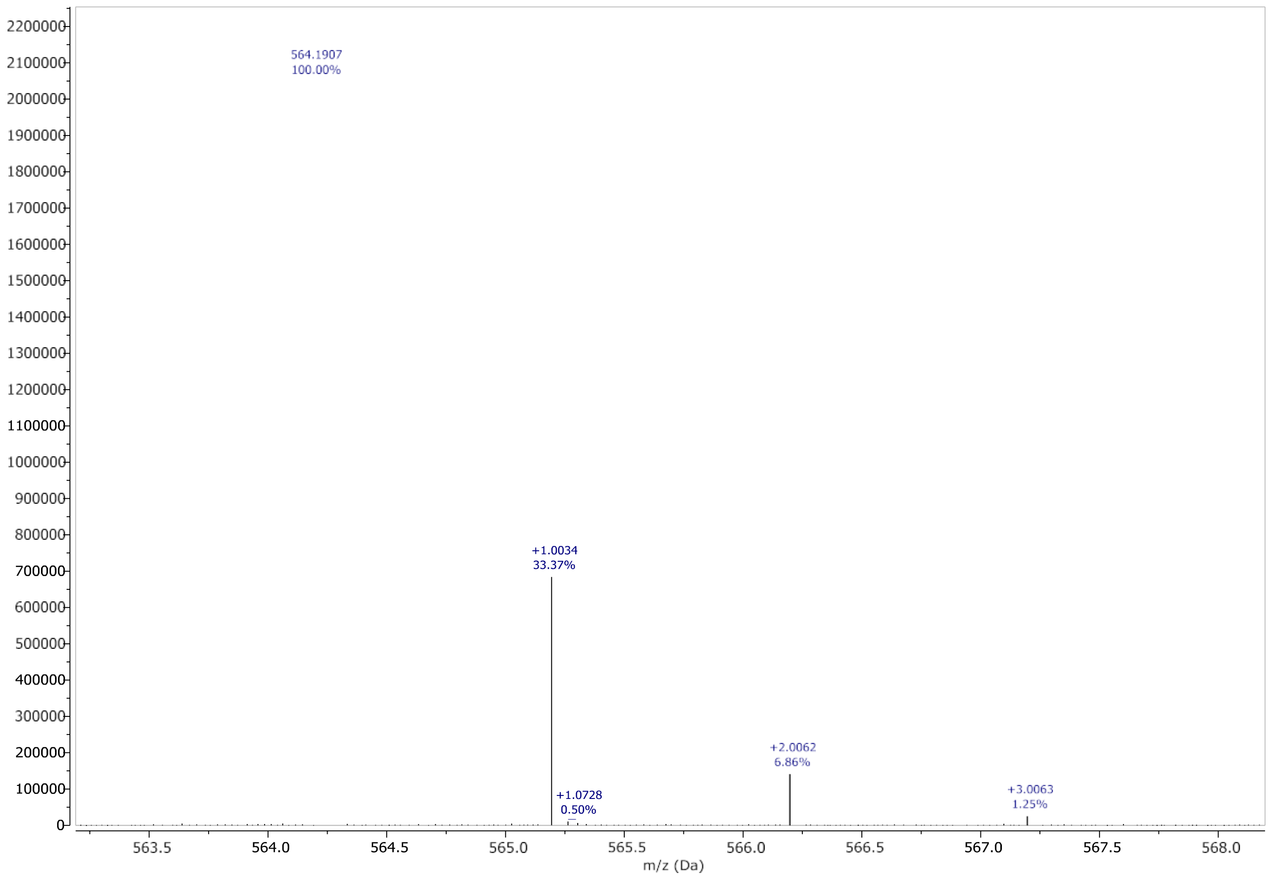

564.1907
100.00%
+1.0034
33.37%
+1.0728
0.50%
+2.0062
6.86%
+3.0063
1.25%
m/z (Da)

5-fluoro-1-((1-(3-((5-hydroxy-3-(4-hydroxyphenyl)-4-oxo-4H-chromen-7-yl)oxy)octyl)-1H-1,2,3-triazol-4-yl)methyl)pyrimidine-2,4(1H,3H)-dione (4f)

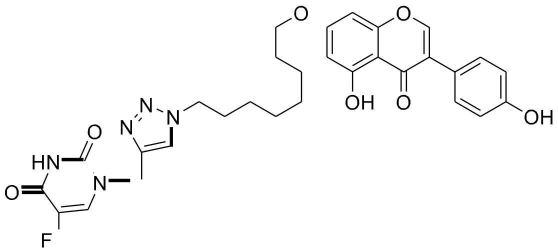

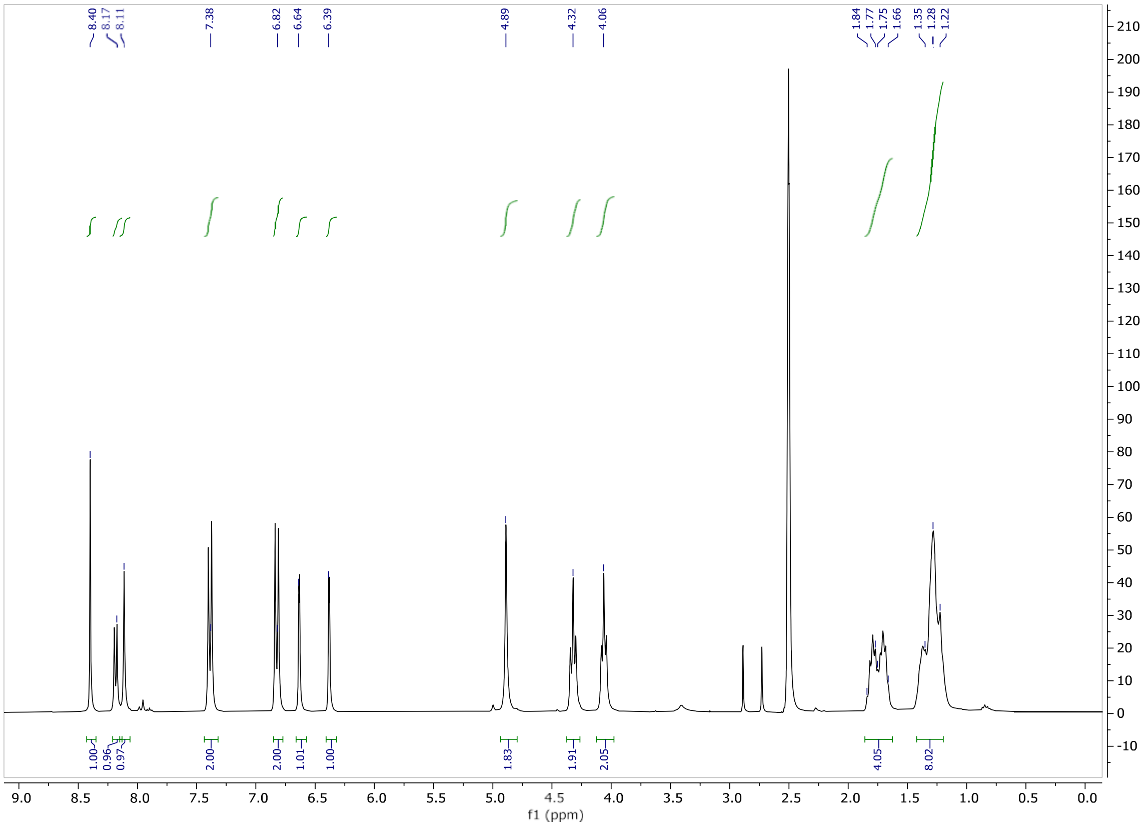

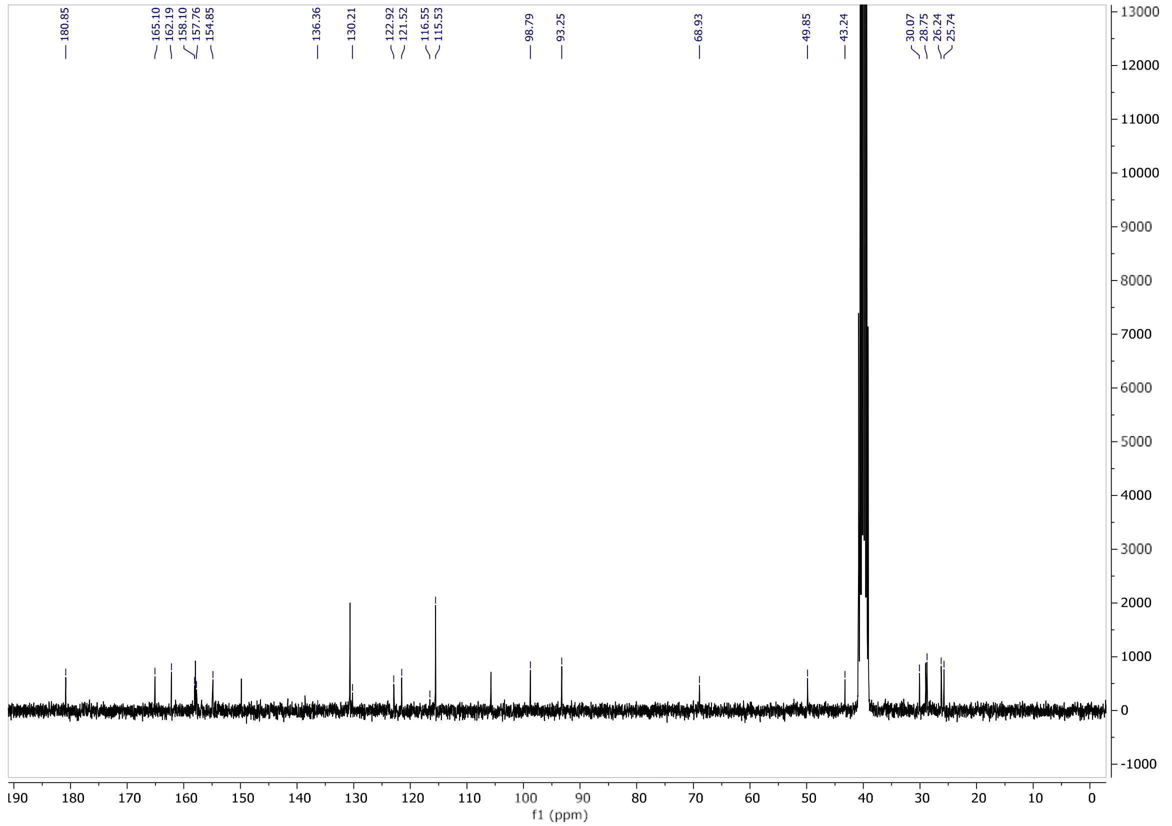

180.85
165.10
162.19
158.10
157.76
154.85
136.36
130.21
122.92
121.52
116.55
115.53
98.79
93.25
68.93
49.85
43.24
30.07
28.75
26.24
25.74
f1 (ppm)

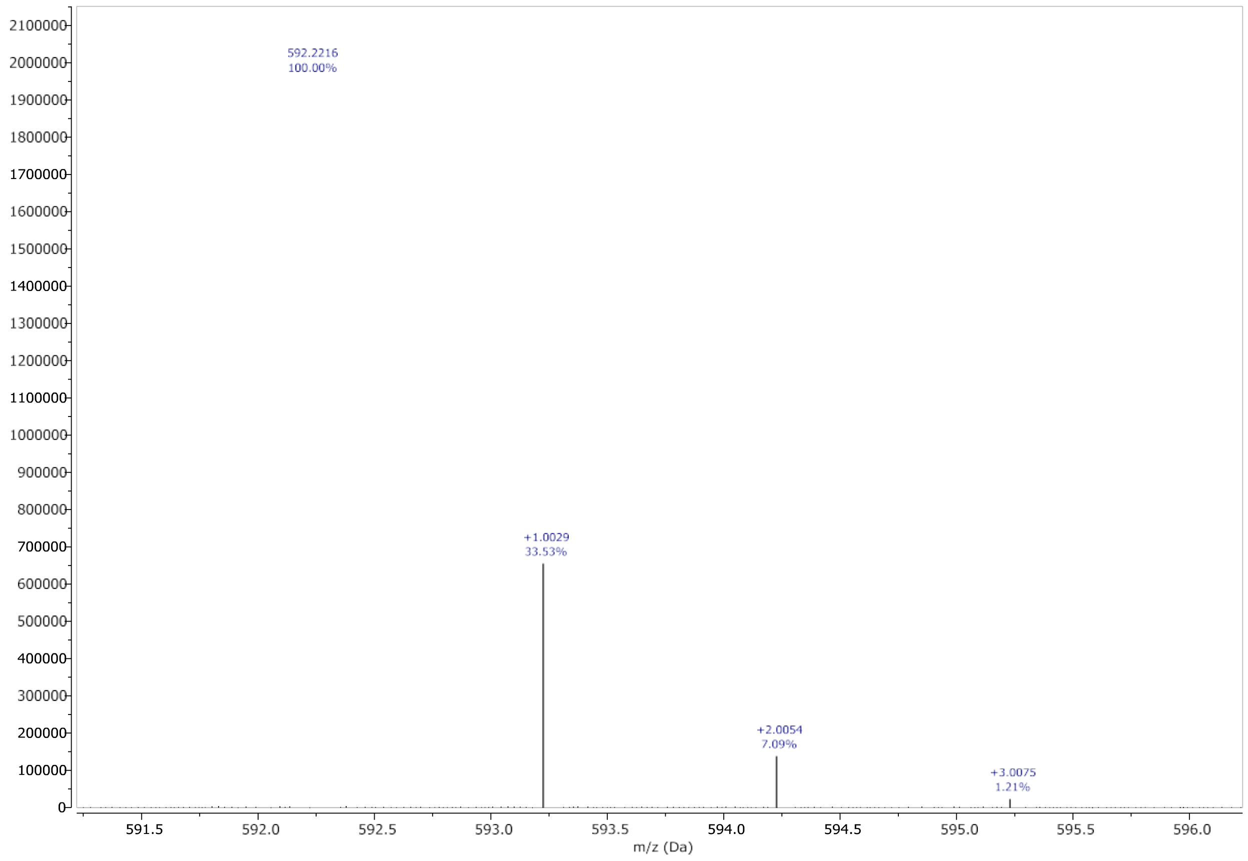

592.2216
100.00%
+1.0029
33.53%
+2.0054
7.09%
+3.0075
1.21%
m/z (Da)

5-fluoro-1-((1-(3-((5-hydroxy-3-(4-hydroxyphenyl)-4-oxo-4H-chromen-7-yl)oxy)nonyl)-1H-1,2,3-triazol-4-yl)methyl)pyrimidine-2,4(1H,3H)-dione (4g)

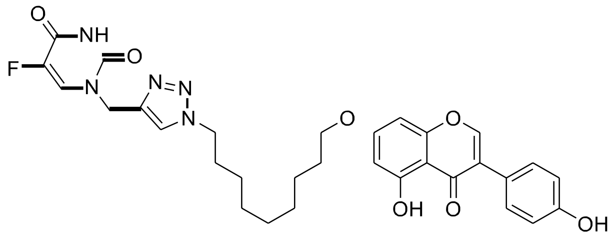

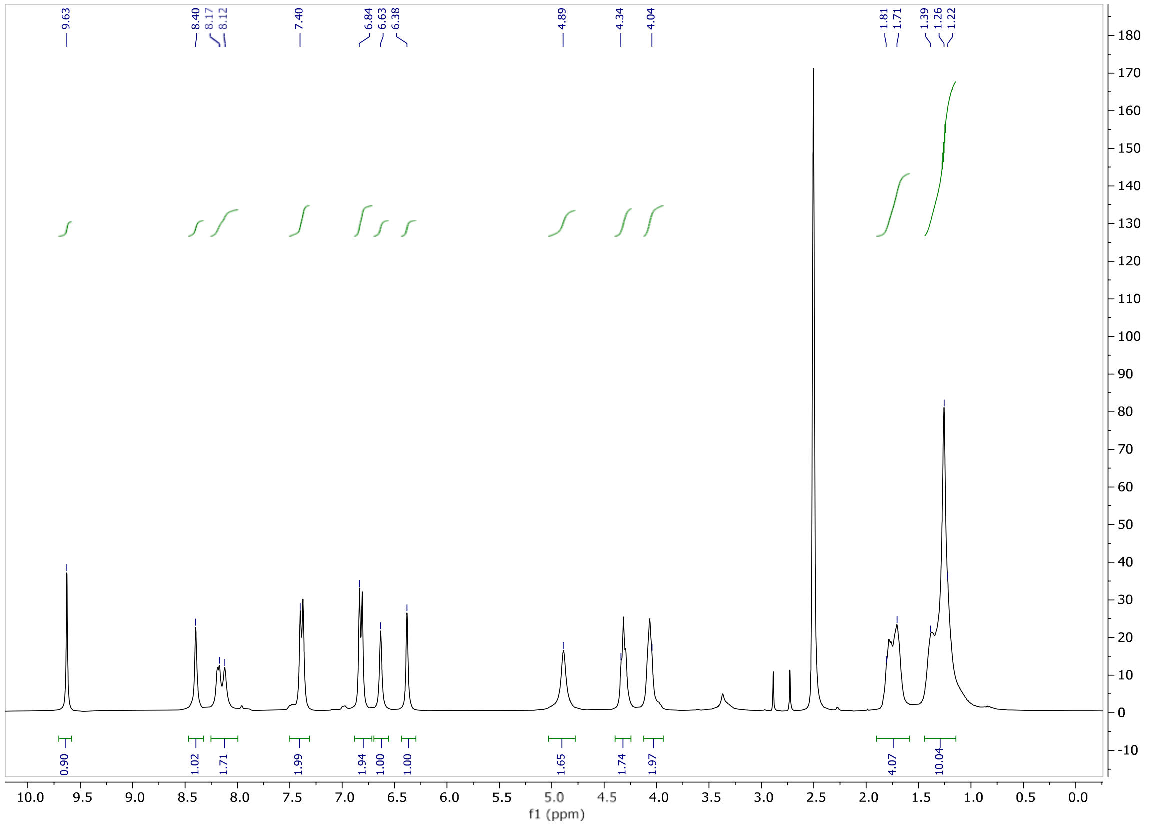

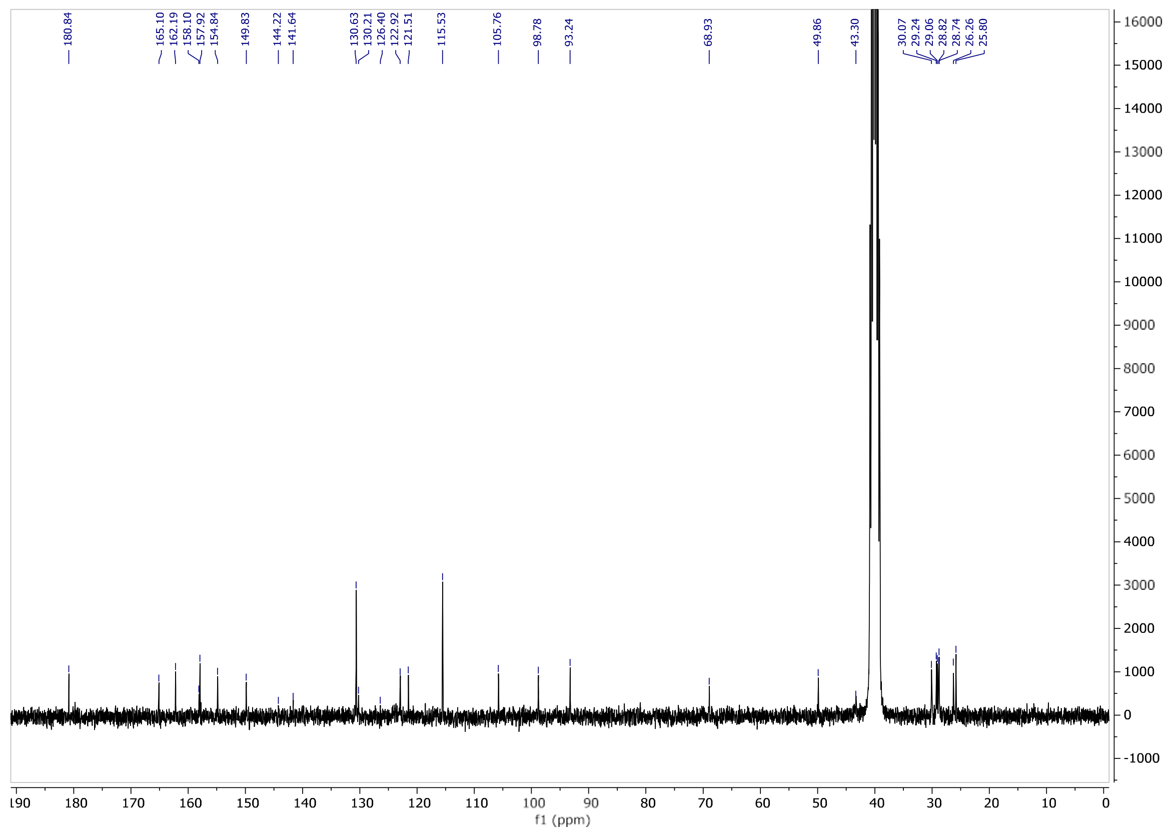

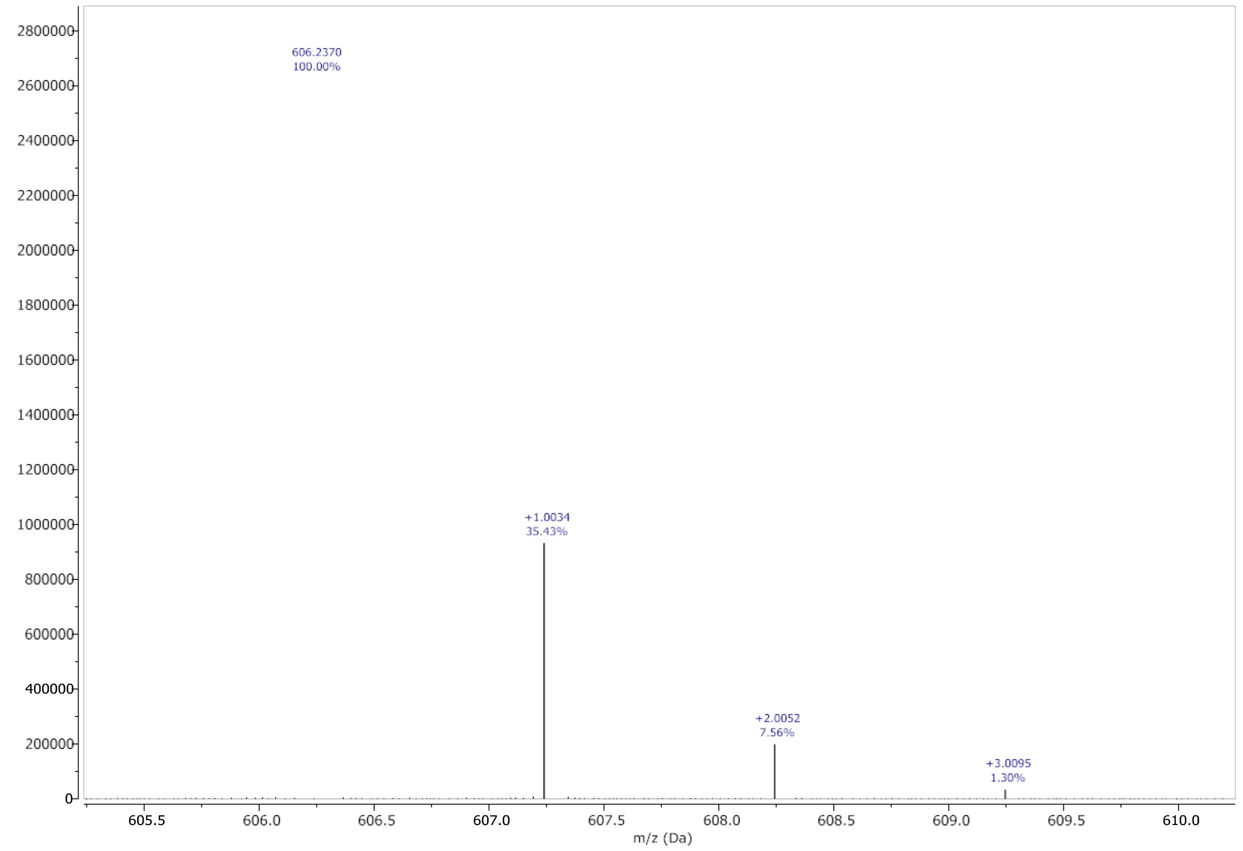

5-fluoro-1-((1-(3-((5-hydroxy-3-(4-hydroxyphenyl)-4-oxo-4H-chromen-7-yl)oxy)dodecyl)-1H-1,2,3-triazol-4-yl)methyl)pyrimidine-2,4(1H,3H)-dione (4h)

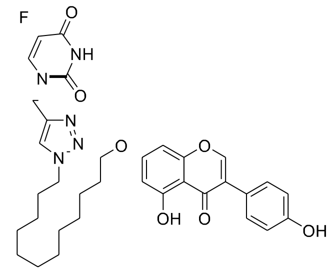

F
O
NH
N
O
N
N
N
O
O
OH
O
OH

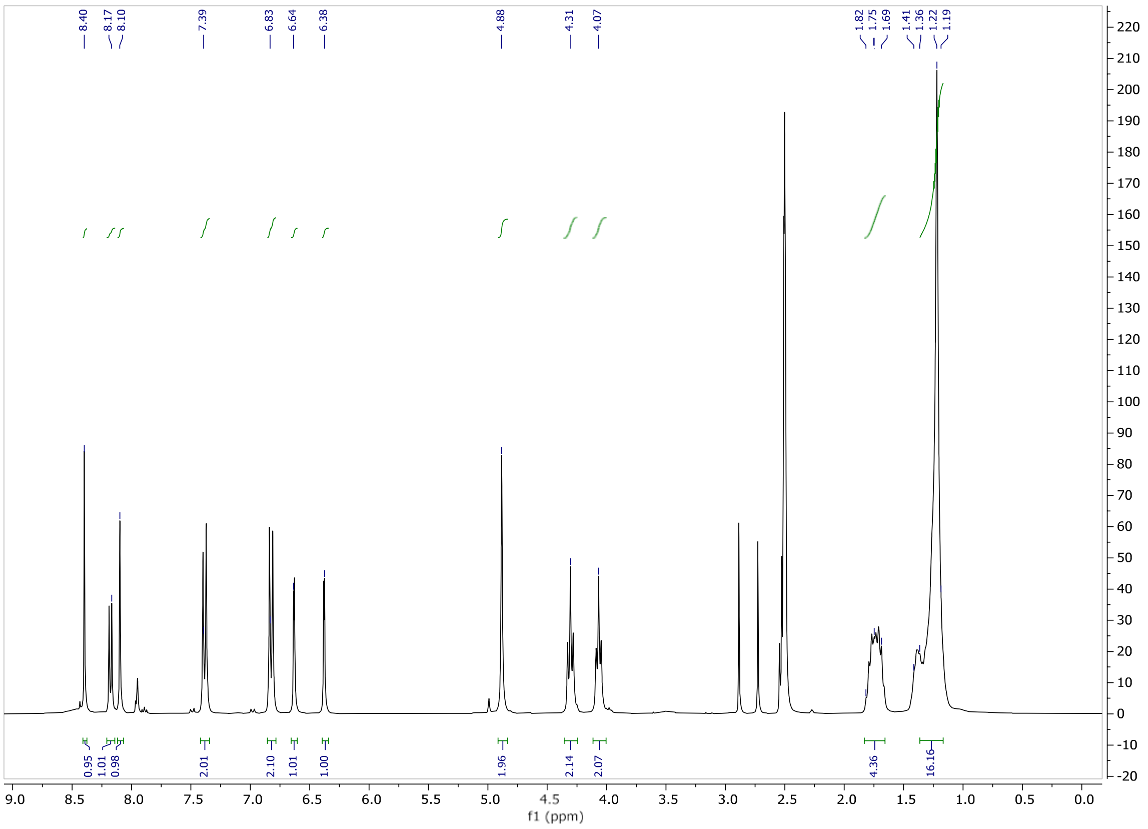

8.40
8.17
8.10
7.39
6.83
6.64
6.38
4.88
4.31
4.07
1.82
1.75
1.69
1.41
1.36
1.22
1.19
0.95
1.01
0.98
2.01
2.10
1.01
1.00
1.96
2.14
2.07
4.36
16.16
f1 (ppm)

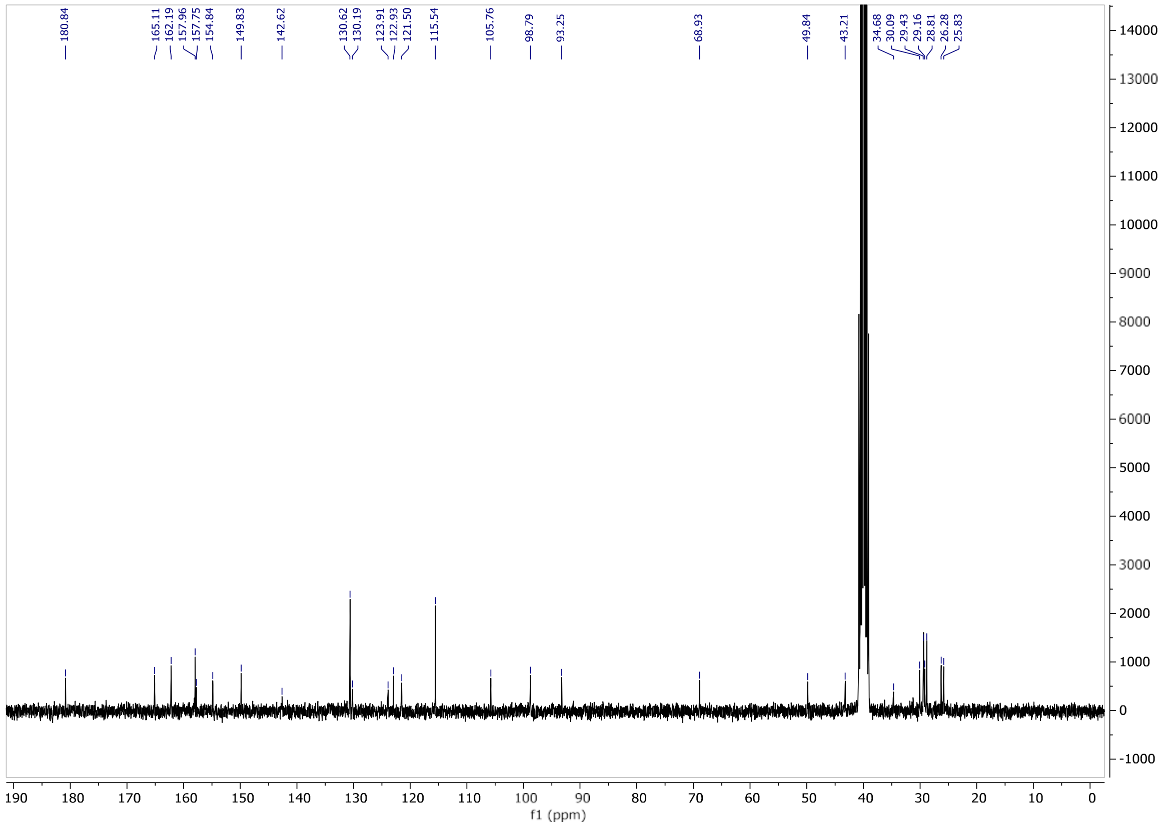
180.84
165.11
162.19
157.96
157.75
154.84
149.83
142.62
130.62
130.19
123.91
122.93
121.50
115.54
105.76
98.79
93.25
68.93
49.84
43.21
34.68
30.09
29.43
29.16
28.81
26.28
25.83
f1 (ppm)

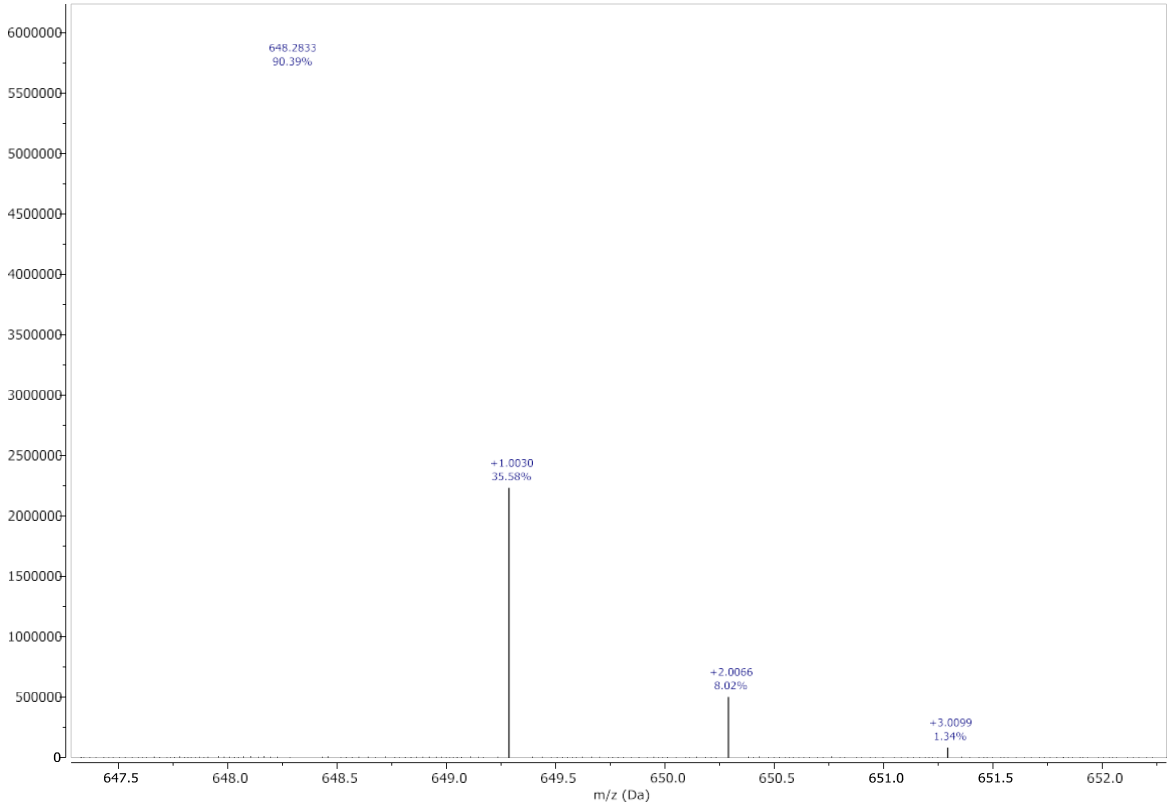
648.2833
90.39%
+1.0030
35.58%
+2.0066
8.02%
+3.0099
1.34%
m/z (Da)

Figure S2. HPLC analysis

Chromatogram and Results

Injection Details

Injectic n Name:	**Muestra 4a**	*Run Time (min):*	**20,00**
Vial Nu mber:	**RA2**	*Injection Volume:*	**20,00**
Injectic n Type:	**Unknown**	*Channel:*	**UV_VIS_1**
Calibra tion Level:		*Wavelength:*	**324**
Instrun ent Method:	**Muestras 130 y 136**	*Bandwidth:*	**n.a.**
Proces sing Method:	**Basic Quantitative**	*Dilution Factor:*	**1,0000**
Injectic n Date/Time:	**30/jun./22 08:31**	*Sample Weight:*	**1,0000**

Chromatogram

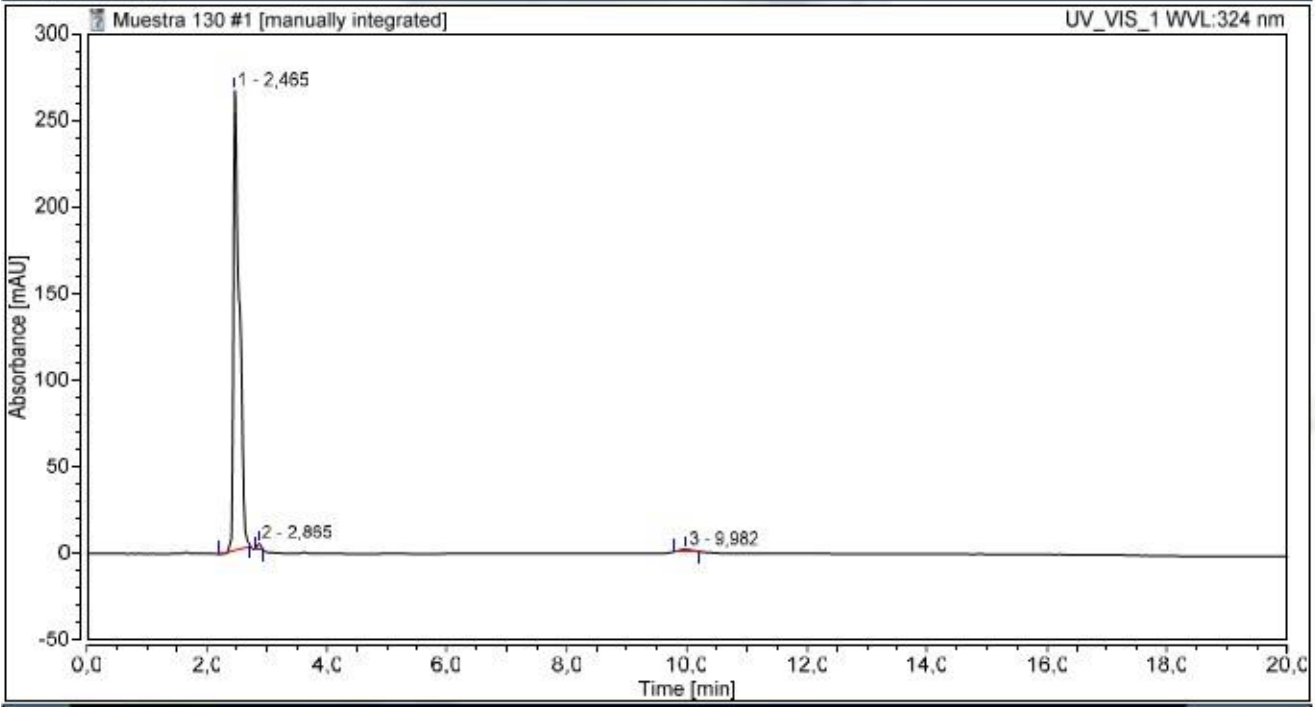

Integration Results

No.	Peak Name	Retention Time min	Area mAU*min	Height mAU	Relative Area %	Relative Height %	Amount n.a.
1		2,465	29,866	265,571	98,46	98,42	n.a.
2		2,865	0,187	3,111	0,62	1,15	n.a.
3		9,982	0,280	1,150	0,92	0,43	n.a.
**Total:**			**30,332**	**269,832**	**100,00**	**100,00**	

Chromatogram and Results

Injection Details

Injection Name:	**4b**	*Run Time (min):*	**12,00**
Vial Number:	**RE3**	*Injection Volume:*	**20,00**
Injection Type:	**Unknown**	*Channel:*	**UV_VIS_1**
Calibration Level:		*Wavelength:*	**324**
Instrument Method:	**Muestras 130 y 136**	*Bandwidth:*	**n.a.**
Processing Method:	**Pureza muestras 130 y 136**	*Dilution Factor:*	**1,0000**
Injection Date/Time:	**30/jun./22 8:49**	*Sample Weight:*	**1,0000**

Chromatogram

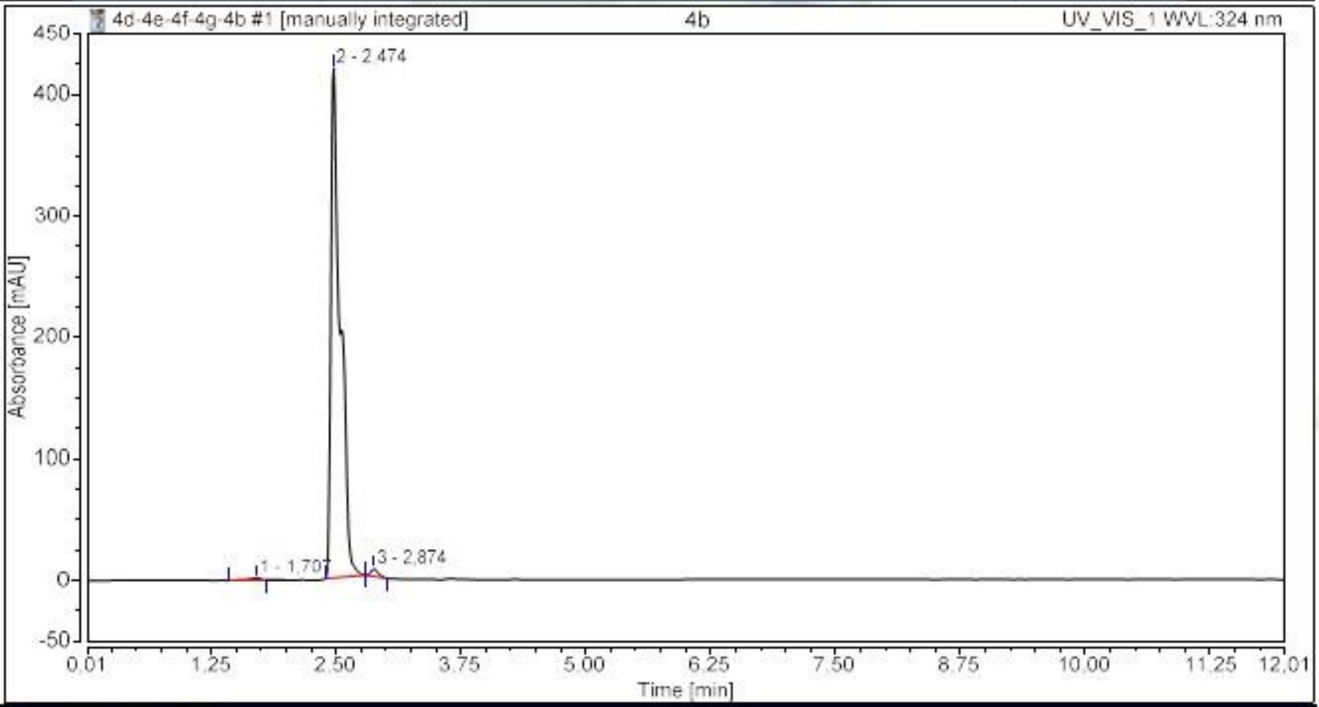

Integration Results

No.	Peak Name	Retention Time<br>min	Area<br>mAU*min	Height<br>mAU	Relative Area<br>%	Relative Height<br>%	Amount<br>n.a.
1		1,707	0,306	2,368	0,65	0,55	n.a.
2		2,474	45,910	420,057	98,19	97,96	n.a.
3		2,874	0,543	6,400	1,16	1,49	n.a.
**Total:**			**46,758**	**428,825**	**100,00**	**100,00**	

Chromatogram and Results

Injection Details

Injection Name:	**4c**	*Run Time (min):*	**12,00**
Vial Number:	**RB2**	*Injection Volume:*	**20,00**
Injection Type:	**Unknown**	*Channel:*	**UV_VIS_1**
Calibration Level:		*Wavelength:*	**324**
Instrument Method:	**Muestras 130 y 136**	*Bandwidth:*	**n.a.**
Processing Method:	**Pureza muestras 130 y 136**	*Dilution Factor:*	**1,0000**
Injection Date/Time:	**30/jun./22 09:16**	*Sample Weight:*	**1,0000**

Chromatogram

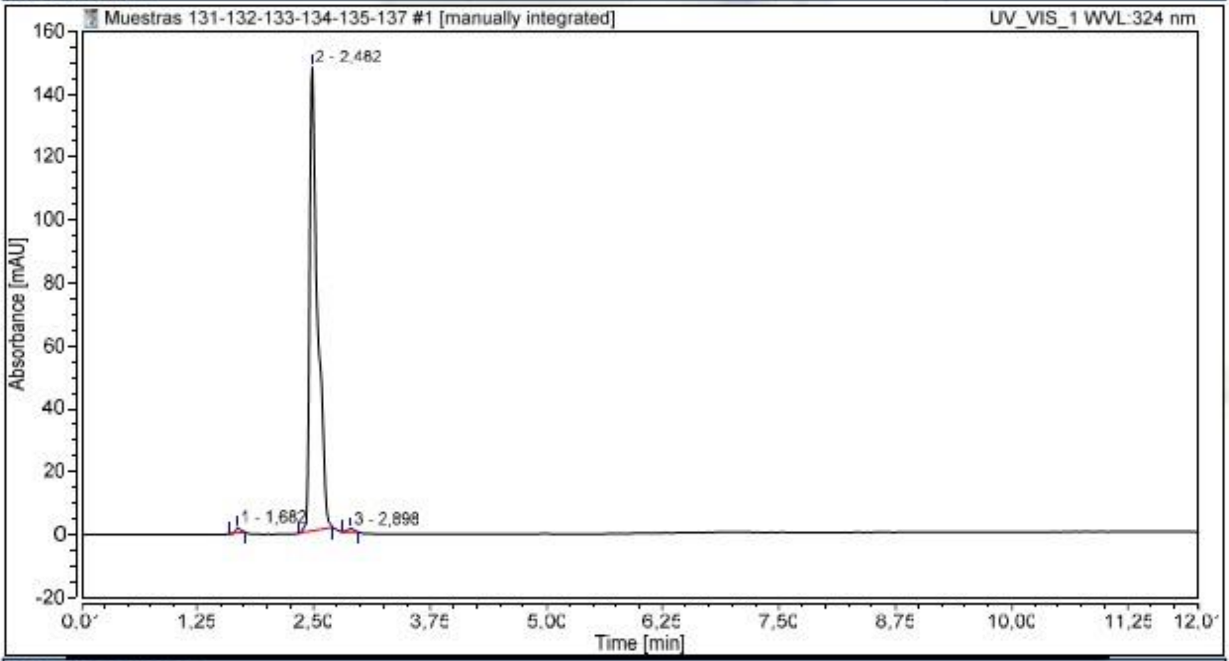

Integration Results

No.	Peak Name	Retention Time min	Area mAU*min	Height mAU	Relative Area %	Relative Height %	Amount n.a.
1		1,682	0,096	1,756	0,65	1,17	n.a.
2		2,482	14,533	147,499	98,77	98,15	n.a.
3		2,898	0,084	1,031	0,57	0,69	n.a.
**Total:**			**14,714**	**150,285**	**100,00**	**100,00**	

Chromatogram and Results

Injection Details

Injection Name:	**4d**	*Run Time (min):*	**10,00**
Vial Number:	**RC1**	*Injection Volume:*	**40,00**
Injection Type:	**Unknown**	*Channel:*	**UV_VIS_1**
Calibration Level:		*Wavelength:*	**324**
Instrument Method:	**Serie 4a-4h_Isocratico_MeOH-ACN_80-20**	*Bandwidth:*	**n.a.**
Processing Method:	**Pureza muestras 130 y 136**	*Dilution Factor:*	**1,0000**
Injection Date/Time:	**06/oct./22 10:01**	*Sample Weight:*	**1,0000**

Chromatogram

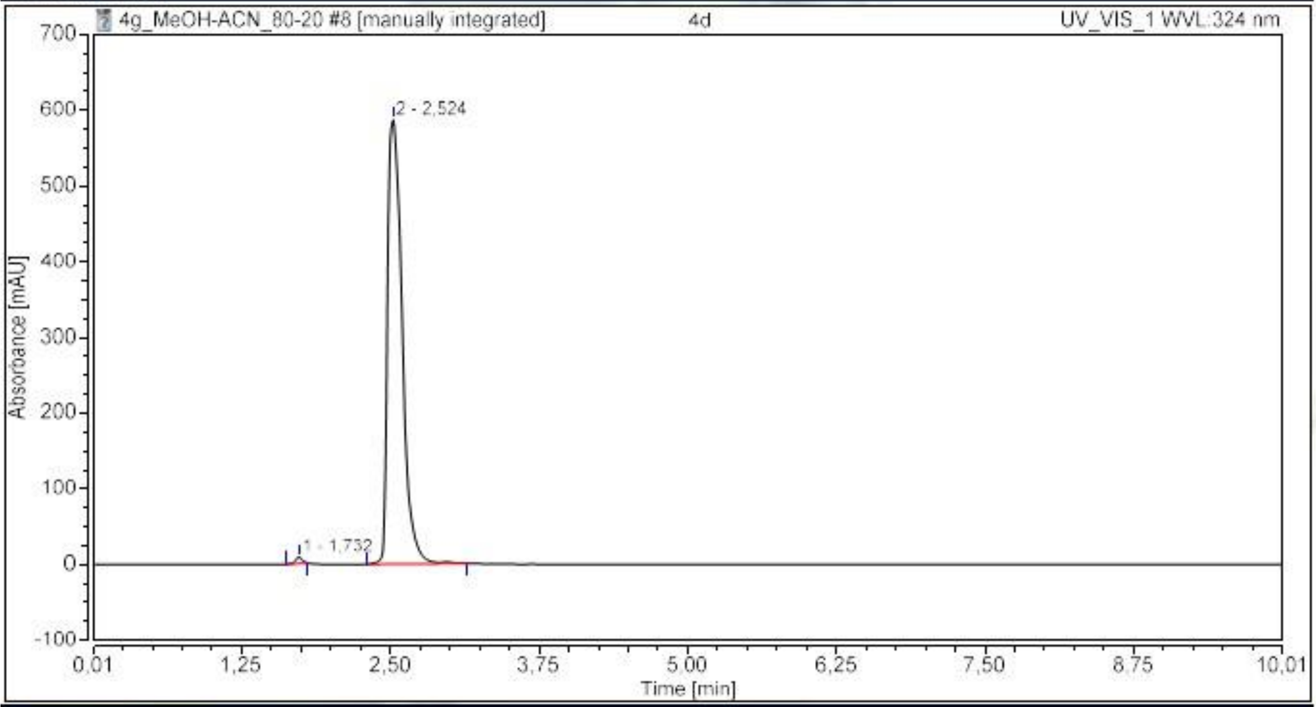

Integration Results

No.	Peak Name	Retention Time min	Area mAU*min	Height mAU	Relative Area %	Relative Height %	Amount n.a.
1		1,732	0,549	8,595	0,65	1,44	n.a.
2		2,524	83,316	586,753	99,35	98,56	n.a.
**Total:**			**83,865**	**595,347**	**100,00**	**100,00**	

Chromatogram and Results

Injection Details

Injectic n Name:	**4e**	*Run Time (min):*	**10,00**
Vial Nι mber:	**RC2**	*Injection Volume:*	**40,00**
Injectic n Type:	**Unknown**	*Channel:*	**UV_VIS_3**
Calibrε tion Level:		*Wavelength:*	**336**
Instrun ent Method:	**Serie 4a-4h_Isocratico_MeOH-ACN_80-20**	*Bandwidth:*	**n.a.**
Proces sing Method:	**Pureza muestras 130 y 136**	*Dilution Factor:*	**1,0000**
Injectic n Date/Time:	**06/oct./22 12:46**	*Sample Weight:*	**1,0000**

Chromatogram

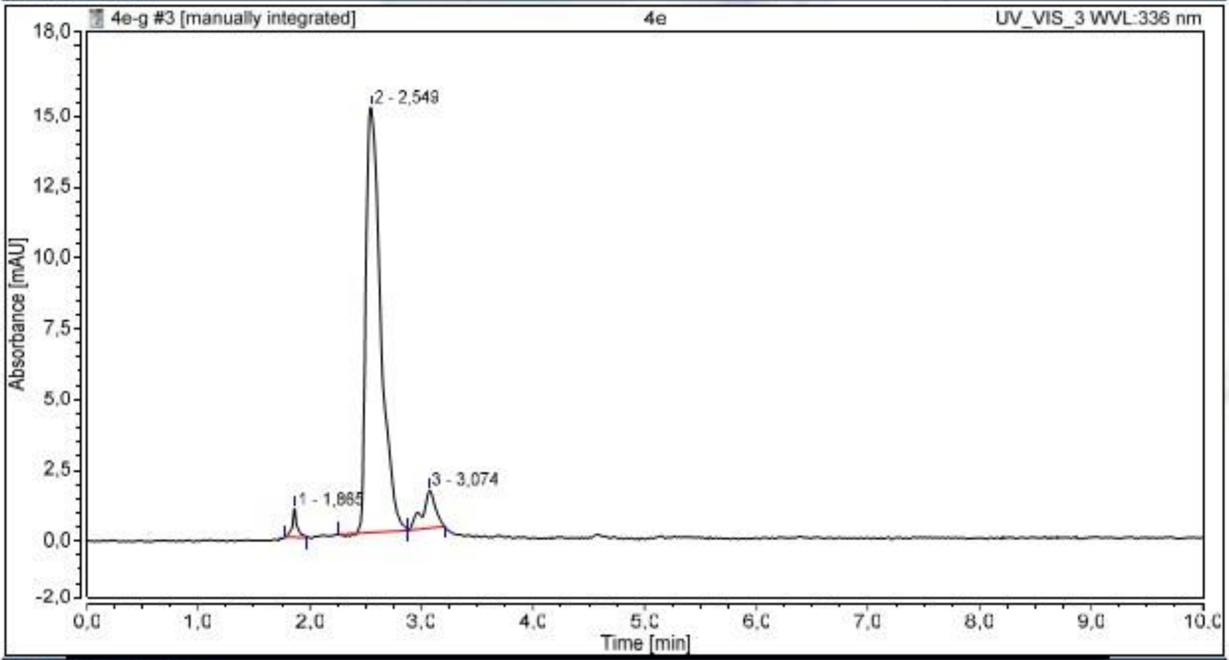

Integration Results

No.	Peak Name	Retention Time<br>min	Area<br>mAU*min	Height<br>mAU	Relative Area<br>%	Relative Height<br>%	Amount<br>n.a.
1		1,865	0,021	0,721	0,86	4,34	n.a.
2		2,549	2,319	15,040	95,85	90,50	n.a.
3		3,074	0,080	0,858	3,29	5,16	n.a.
**Total:**			**2,419**	**16,619**	**100,00**	**100,00**	

Chromatogram and Results

Injection Details

Injection Name:	**4f**	*Run Time (min):*	**10,00**
Vial Number:	**RC3**	*Injection Volume:*	**40,00**
Injection Type:	**Unknown**	*Channel:*	**UV_VIS_4**
Calibration Level:		*Wavelength:*	**263**
Instrument Method:	**Serie 4a-4h_Isocratico_MeOH-ACN_80-20**	*Bandwidth:*	**n.a.**
Processing Method:	**Pureza muestras 130 y 136**	*Dilution Factor:*	**1,0000**
Injection Date/Time:	**06/oct./22 10:45**	*Sample Weight:*	**1,0000**

Chromatogram

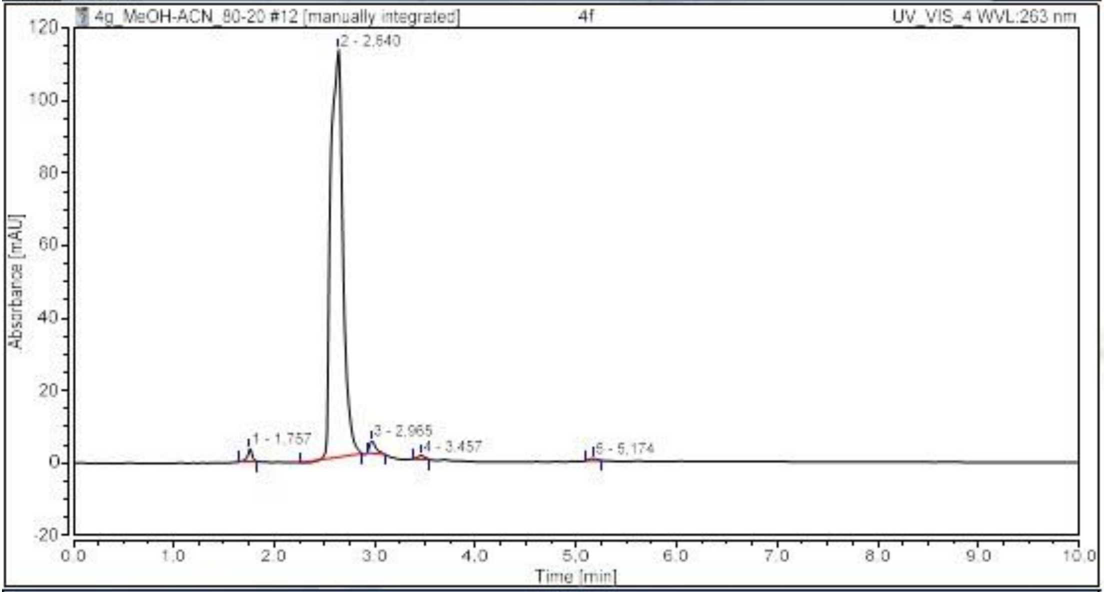

Integration Results

No.	Peak Name	Retention Time min	Area mAU*min	Height mAU	Relative Area %	Relative Height %	Amount n.a.
1		1,757	0,174	3,460	1,07	2,87	n.a.
2		2,640	15,736	112,341	96,62	93,17	n.a.
3		2,965	0,248	3,287	1,53	2,73	n.a.
4		3,457	0,086	0,993	0,53	0,82	n.a.
5		5,174	0,042	0,493	0,26	0,41	n.a.
**Total:**			**16,287**	**120,575**	**100,00**	**100,00**	

Chromatogram and Results

Injection Details

Injection Name:	**4g**	*Run Time (min):*	**10,00**
Vial Number:	**RE3**	*Injection Volume:*	**40,00**
Injection Type:	**Unknown**	*Channel:*	**UV_VIS_4**
Calibration Level:		*Wavelength:*	**263**
Instrument Method:	**Serie 4a-4h_Isocratico_MeOH-ACN_80-20**	*Bandwidth:*	**n.a.**
Processing Method:	**Pureza muestras 130 y 136**	*Dilution Factor:*	**1,0000**
Injection Date/Time:	**06/oct./22 14:17**	*Sample Weight:*	**1,0000**

Chromatogram

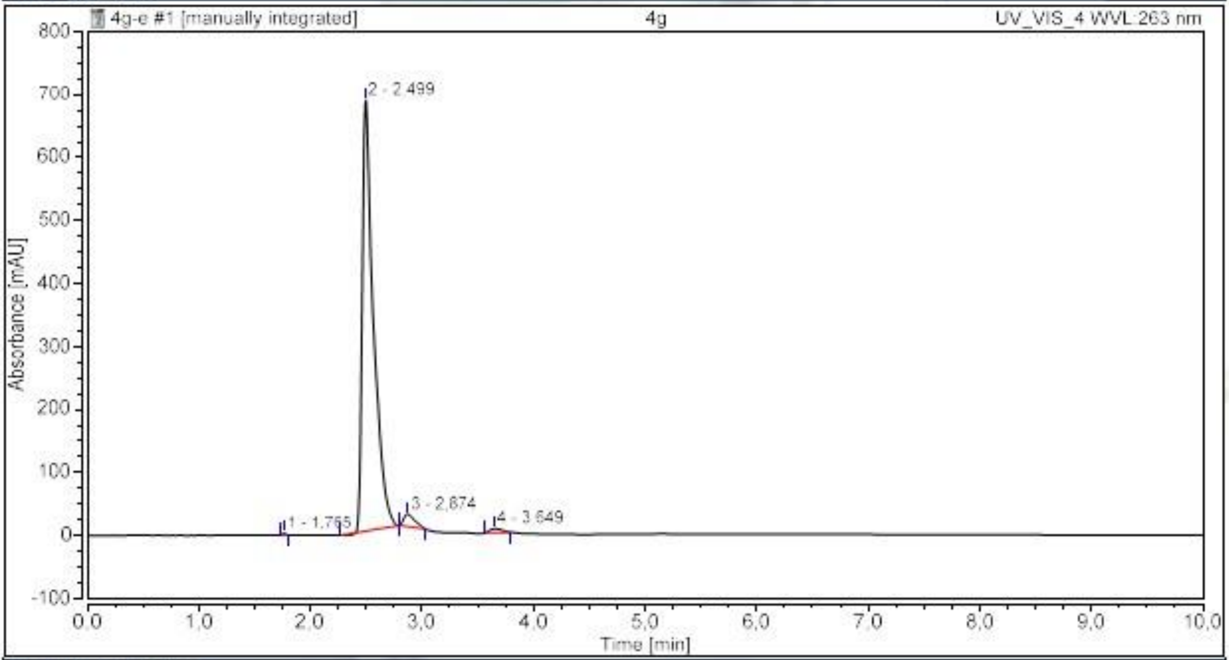

Integration Results

No.	Peak Name	Retention Time min	Area mAU*min	Height mAU	Relative Area %	Relative Height %	Amount n.a.
1		1,765	0,104	3,151	0,13	0,44	n.a.
2		2,499	79,204	685,067	96,10	95,87	n.a.
3		2,874	2,320	20,139	2,81	2,82	n.a.
4		3,649	0,794	6,232	0,96	0,87	n.a.
**Total:**			**82,421**	**714,589**	**100,00**	**100,00**	

Chromatogram and Results

Injection Details

Injectic n Name:	**Muestra 4h**	*Run Time (min):*	**20,00**
Vial N mber:	**RA3**	*Injection Volume:*	**20,00**
Injectic n Type:	**Unknown**	*Channel:*	**UV_VIS_1**
Calibr: tion Level:		*Wavelength:*	**324**
Instrur. ent Method:	**Muestras 130 y 136**	*Bandwidth:*	**n.a.**
Proces sing Method:	**Pureza muestras 130 y 136**	*Dilution Factor:*	**1,0000**
Injectic n Date/Time:	**30/jun./22 11:01**	*Sample Weight:*	**1,0000**

Chromatogram

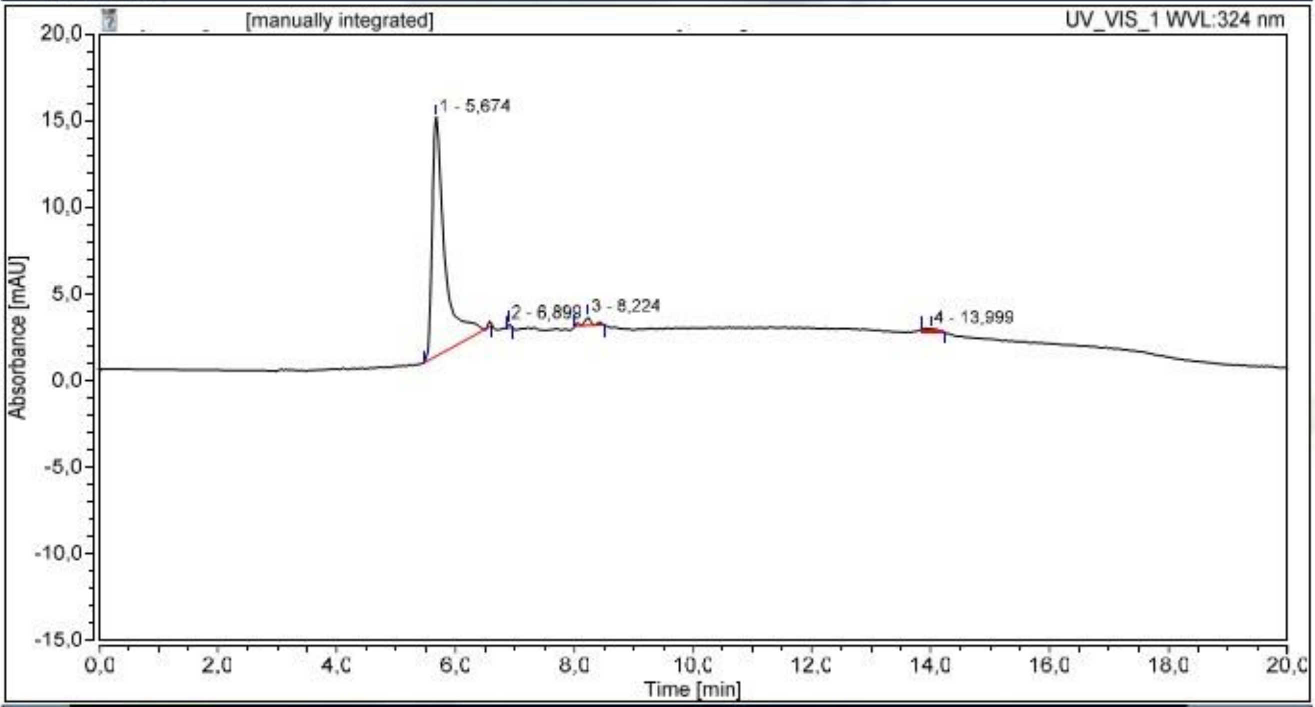

Integration Results

No.	Peak Name	Retention Time<br>min	Area<br>mAU*min	Height<br>mAU	Relative Area<br>%	Relative Height<br>%	Amount<br>n.a.
1		5,674	3,433	13,811	96,79	93,93	n.a.
2		6,899	0,013	0,235	0,36	1,60	n.a.
3		8,224	0,072	0,496	2,02	3,37	n.a.
4		13,999	0,029	0,162	0,83	1,10	n.a.
**Total:**			**3,547**	**14,704**	**100,00**	**100,00**	
